# Intact in vivo visualization of telencephalic microvasculature in medaka using optical coherence tomography

**DOI:** 10.1038/s41598-020-76468-6

**Published:** 2020-11-16

**Authors:** Takashi Suzuki, Tomohiro Ueno, Naoya Oishi, Hidenao Fukuyama

**Affiliations:** 1 Center for Health Science Innovation, Osaka City University, Osaka, Japan; 2 Department of Psychiatry, Kyoto University Hospital, Kyoto, Japan; 3 Human Health Sciences, Graduate School of Medicine, Kyoto University, Kyoto, Japan; 4 Medical Innovation Center, Graduate School of Medicine, Kyoto University, Kyoto, Japan; 5 Kyoto University, Kyoto, Japan

**Keywords:** Optical imaging, Experimental models of disease, Imaging and sensing

## Abstract

To date, various human disease models in small fish—such as medaka (*Oryzias lapties*)—have been developed for medical and pharmacological studies. Although genetic and environmental homogeneities exist, disease progressions can show large individual differences in animal models. In this study, we established an intact in vivo angiographic approach and explored vascular networks in the telencephalon of wild-type adult medaka using the spectral-domain optical coherence tomography. Our approach, which required neither surgical operations nor labeling agents, allowed to visualize blood vessels in medaka telencephala as small as about 8 µm, that is, almost the size of the blood cells of medaka. Besides, we could show the three-dimensional microvascular distribution in the medaka telencephalon. Therefore, the intact in vivo imaging via optical coherence tomography can be used to perform follow-up studies on cerebrovascular alterations in metabolic syndrome and their associations with neurodegenerative disease models in medaka.

## Introduction

Metabolic syndrome is known to be associated with an increased risk of cardiovascular death^1^, stroke^2^, age-related cognitive decline^3^, and dementia^4,5^. Nonetheless, the relationship between metabolic syndrome, cerebrovascular disorders, and cognitive functions is not fully understood. Since cognitive functions are mainly originated from the cerebrum/telencephalon, non-invasive in vivo angiography in animal disease models is crucial for understanding the underlying mechanisms and developing effective treatments.

Medaka (*Oryzias lapties*) and zebrafish (*Danio rerio*) are small freshwater fish and space- and cost-effective animal models from Vertebrata. Particularly, the medaka has become an excellent model organism for developmental biology and genetics^6^. Sequencing of the medaka genome has been completed and techniques for producing transgenic and knockout animals have been established^7,8^. Due to the remarkable advances in genome-editing techniques, various human disease models in medaka, such as the tumor suppressor gene P53 knockout model^9^ and the neuropathic Gaucher disease model^10^, have been generated. Interestingly, there are metabolic similarities between medaka and humans: for instance, feeding medaka with a high-fat diet can lead to the development of non-alcoholic steatohepatitis (NASH)^11^—a progressive form of non-alcoholic fatty liver disease (NAFLD)^12^—and obesity-related glomerulopathy (ORG)^13^, which mimic human metabolic syndrome. Although there are several inbred medaka strains with genetic and environmental similarities, these diseases occur and develop heterogeneously in each fish^9–11^.

In vivo cerebrovascular imaging in small fish models following disease development could be useful to understand mechanisms between metabolic syndrome and cerebral disorders. Due to their transparency, the early developmental stages of these fish can be observed with stereo and confocal microscopy. In fact, the morphogenesis of optic tectum^14^ and cerebellum^15^ has been investigated in detail. Cerebrovascular development in zebrafish from the post-hatching to the adult stage has been studied with confocal and multiphoton laser-scanning microscopy using green fluorescent protein markers^16^. Since the metabolic syndrome develops at the post-embryonic stage, these techniques may not be applicable to the disease models due to the lack of body transparency. Administration of a fluorescent label or contrast agent is usually required for optical imaging techniques such as confocal fluorescence microscopy and two-photon microscopy at the adult stage. Nevertheless, applying these agents to small fish is not only complex and time-consuming but may also change the natural physiological processes of fish. Therefore, another in vivo imaging technique would be necessary to thoroughly investigate cerebrovascular changes and disease progression.

Magnetic resonance imaging (MRI) enables the non-invasive visualization of spatial distributions of various physical structures and chemical properties of internal organs. A high-resolution kind of MRI, the MR microscopy, has been used to study in detail the anatomical structures in adult zebrafish^17^ and to evaluate the hepatic steatosis levels in NAFLD-affected adult medaka over time^18^. The MR microscopy is suitable to study various development processes in intact opaque adult fish non-invasively. Besides the MR microscopy, an X-ray-based micro-computed tomography^19^ and an ultrasound-based bio-microscopy^20^ have also been implemented in zebrafish-model studies. Nonetheless, whereas X-ray has inevitable ionizing radiation and limited sensitivity to soft tissues, the low contrast and speckle artifact of ultrasound imaging prevent its extensive applications in the zebrafish-model studies. However, these imaging techniques do not have sufficiently high enough spatial resolution to show features in small cerebral blood vessels of the fish.

Optical coherence tomography (OCT) is a non-invasive technique based on near-infrared light and capable to provide cross-sectional and three-dimensional images of light-scattering media, such as living tissues. In fact, those are better penetrated by near-infrared light compared to visible light due to their low scatterance and absorbance at longer wavelengths. However, this effect diminishes at wavelengths longer than 950 nm owing to increased absorption by water and lipids; nonetheless, a clear window exists at wavelengths between 650 and 950 nm, thus enabling optical imaging in living animals^21,22^. The spectral-domain OCT (SD-OCT) systems allow for resolving structures down to a few micrometers in the axial direction^23,24^, while the utilization of microscopic objectives provides micrometric resolution in the lateral direction. The high speed of the SD-OCT systems allowed not only to obtain structural tissue images and information, but also to investigate the blood flow in vivo^25^. These properties have been exploited for several studies on small aquatic animals, such as morphological studies on zebrafish^26,27^ and medaka^28^, as well as for investigating the cardiovascular development in *Xenopus*^29^. In addition to morphological studies, OCT angiography (OCTA) permits the visualization of functional blood vessels in living tissues by exploiting the variation in the OCT signal caused by moving particles such as red blood cells^30,31^. Furthermore, Doppler OCT was developed to measure blood flow velocity in microvessels with a spatial resolution of a few micrometers. Doppler-based OCTA is also widely used to characterize the cerebrovascular network of rodents^32,33^, albeit a cranial window is required as optical access in such models. On the other hand, the adult medaka maintains OCT-suitable transparency of body skin to some extent compared with other animal models; additionally, the smaller size of its brain enables the visualization of deep structures. To date, only a few studies focused on cerebrovascular OCT imaging in medaka older than one year. Concerning cognitive functions, neuronal and molecular mechanisms for behaviors such as mating have been found in medaka^34,35^; thus, behavioral differences due to cerebral vasculature alterations can be investigated in OCT-visualized medaka.

In this study, we performed intact in vivo angiographic imaging of the telencephalic microvasculature in adult wild-type medakas using SD-OCT in order to facilitate individual follow-up studies on disease progression in metabolic syndrome-related disease medaka models.

## Results

### Intact visualization of medaka telencephalon by OCT

We developed a special sample-manipulation procedure to visualize microvessels in vivo in medaka telencephalon using OCT. We implemented the sample-manipulation procedure for seven adult medakas, and we were able to visualize in vivo telencephalons in all seven adult medaka using OCT. During an in vivo imaging session of about 20 min, no medaka did show visually conceivable body movements. After the in vivo imaging, all seven adult medakas were returned to a recovery water tank, restarted opercular movements, and then recovered to a normal swimming condition in about 30 min.

**Figure 1:**
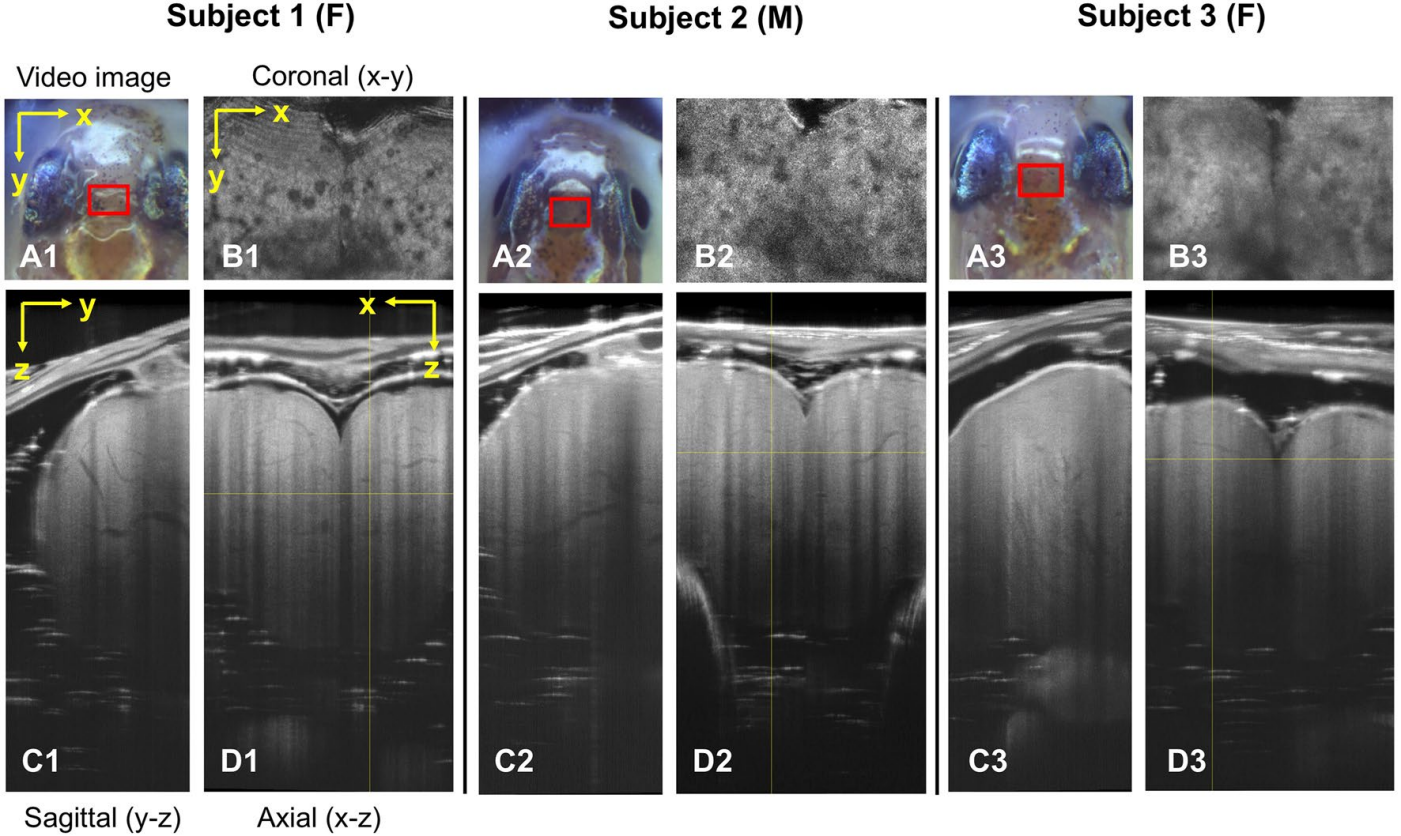
Cross-sectional OCT images of three medaka telencephala. (**A1–3**) Video camera images of Subjects 1–3 (ThorImage®OCT v4.4, https://www.thorlabs.com/newgrouppage9.cfm?objectgroup_id=7982). The red rectangles indicate the scan areas. (**B1–3**) Coronal (x–y plane), (**C1–3**) sagittal (y–z plane), and (**D1–3**) axial (x–z plane) section images of the medaka telencephalon are shown along the yellow lines in D in Subjects 1–3 (ImageJ 1.52n, https://imagej.nih.gov/ij). Darker thread-like branched structures are observable. The yellow arrows in the OCT image of Subject 1 indicate the axis of OCT scan coordinates. The sizes of images are x: 0.95 mm, y: 0.70 mm, and z: 1.91 mm. F: female, M: male.

Representative sets of OCT orthogonal cross-sectional planes from three medakas are shown in Fig. 1. Each cross-sectional image underwent a perpendicular Gaussian filtering process. The regions of the three images correspond to red squares in the video images. The sagittal (i.e., y–z plane) and axial (i.e., x–z plane) sectional OCT images showed, from top to bottom (i.e., along the z-direction), the skin, skull, pia mater, and telencephalon. In each cross-section, thread-like branching structures darker than the surrounding elements and continuously connected in each slice direction were observed in the telencephala (Supplementary Movie 1: Media 1 and Media 2). Media 1 shows the axial plane from anterior to posterior, whereas Media 2 shows the sagittal plane from left to right. The darker thread-like structures tended to become thinner as they extended to the anterior and lateral sides of the telencephalon. Besides, at the dorsal side of the telencephalon, most of these darker thread-like structures were thin. Collectively, these darker thread-like structures showed the characteristics of blood vessels of medaka. On the coronal (i.e., x–y plane) section, some large dark circles with a diameter of 10–30 μm were observed (Fig. 1B). These parts appeared in almost all slices without changing their sizes. In the sagittal and axial sections, they corresponded to dark band structures extending along the z-direction. On the other hand, some other small darker elements with a diameter of about 12 μm changed their position continuously as the slice moved ventrally along the z-direction (Supplementary Movie 1: Media 3). Media 3 shows the coronal plane from the top to the bottom of the head. Therefore, these dark elements corresponded to the darker thread-like structures in the sagittal and axial sections.

### Morphological confirmation of blood vessels in medaka telencephalon

**Figure 2:**
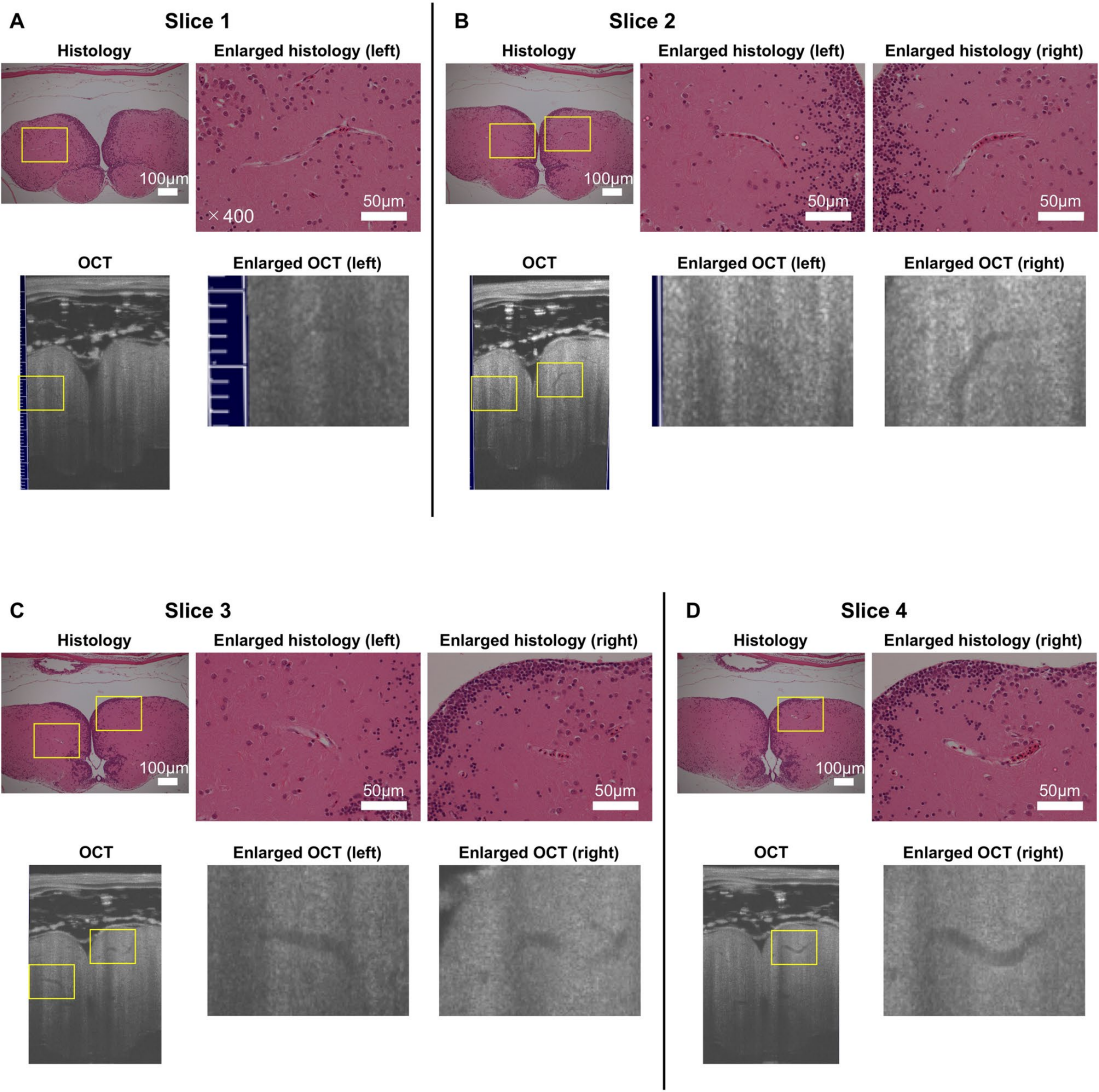
Anatomical correspondence between histology and OCT images. The histology and the corresponding OCT images from Subject 6 (female) are shown in the upper and lower rows, respectively (ImageJ 1.52n, https://imagej.nih.gov/ij). The yellow squares indicate representative correspondences. The enlarged views of the yellow squares are shown next to the original histology and OCT images. The column of the enlarged views corresponds to a lateral position in the brain [(**A**), left; (**B**), left, right; (**C**), left, right; (**D**), right]. Cutting positions are approximately 45 [(**A**), Slice 1], 93 [(**B**), Slice 2], 114 [(**C**), Slice 3], and 120 [(**D**), Slice 4] μm from the tip of the cerebrum. The histological sections were stained with hematoxylin and eosin. The white bars in the original and enlarged histology images correspond to 100 and 50 μm, respectively.

To confirm the vascular nature of the darker thread-like structures in the telencephalon, we compared the OCT images with histological specimens from the same subjects (Fig. 2). The upper row of Fig. 2 shows hematoxylin–eosin-stained axial sections of a medaka brain; a few large blood vessels with nucleated red blood cells are observable therein. Further, high-magnification images of the corresponding histological specimens are shown next to the original histology; endothelial cells surrounding nucleated red blood cells are observable therein. Since the cutting axis of the histological serial sections did not perfectly match the sagittal (y) axis of the 3D OCT images, we looked for the same histological features in an arbitrary slice of the OCT image. In the lower row of Fig. 2, corresponding arbitrary slices of the original and enlarged OCT images of the corresponding regions are shown. The darker thread-like structures had the same features of blood vessels in the corresponding high-magnification histology. However, the shape of the brain tissue specimen was wider than that of the OCT image; besides, the relative size of the telencephalon was smaller and the distance between the pia mater and the telencephalon was larger in the histology than in the OCT image. Although these differences in shape and size existed, in the OCT images we could find the similar features of blood vessels that could be seen in histology.

### Functional verification of blood vessels in medaka telencephalon

**Figure 3:**
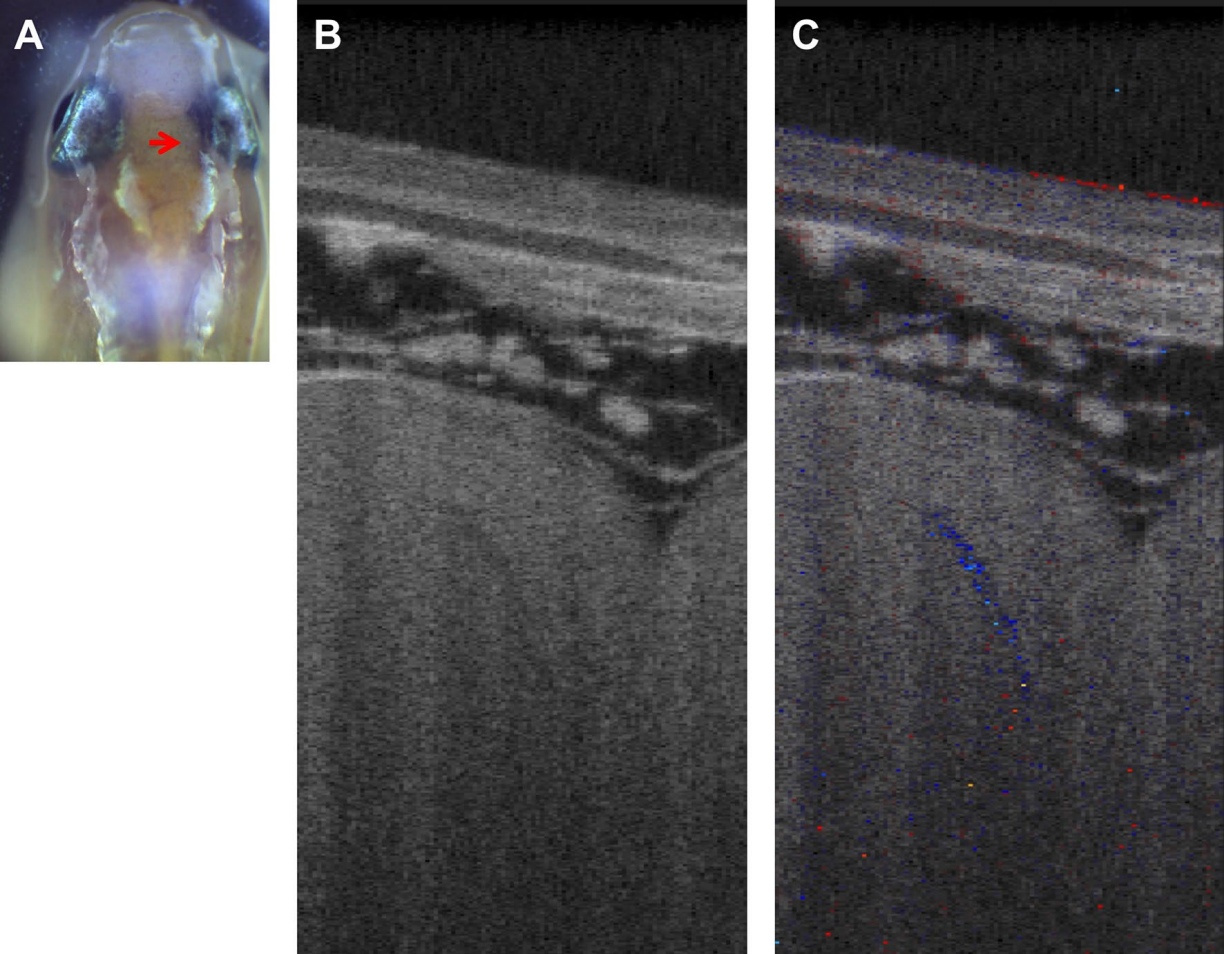
Verification of blood flow by Doppler OCT. (**A**) Video camera image (ThorImage®OCT v4.4, https://www.thorlabs.com/newgrouppage9.cfm?objectgroup_id=7982). The red arrow indicates the scan line and area (0.5 mm) of OCT. (**B**) 2D OCT image and (**C**) corresponding Doppler OCT image of the medaka telencephalon (ImageJ 1.52n, https://imagej.nih.gov/ij). The darker thread-like structure in (**B**) appears in blue in (**C**), indicating the presence of a Doppler frequency shift induced by a backward flow.

In addition to the morphological confirmation, we performed functional verification that the darker thread-like structures in the OCT images had blood flow by using Doppler OCT, which can provide quantitative information on intravascular flow rate in vivo. Figure 3 shows a representative set comprising a 2D OCT image and its corresponding Doppler OCT image. The image plane is indicated by a red arrow in the video image (Fig. 3A). Only the darker thread-like telencephalic structure in the 2D OCT image (Fig. 3B) got colored in the Doppler OCT image (Fig. 3C) due to the Doppler effect. In this case, the blue color indicates the backward direction of the flow relatively to the OCT beam. The real-time measurement of the Doppler OCT (Supplementary Movie 2: Media 4) showed fluctuations of the Doppler shifts at the darker thread structure in Fig. 3B, yet another indication of the presence of blood flow therein.

### Measurement of blood vessel diameters

**Figure 4:**
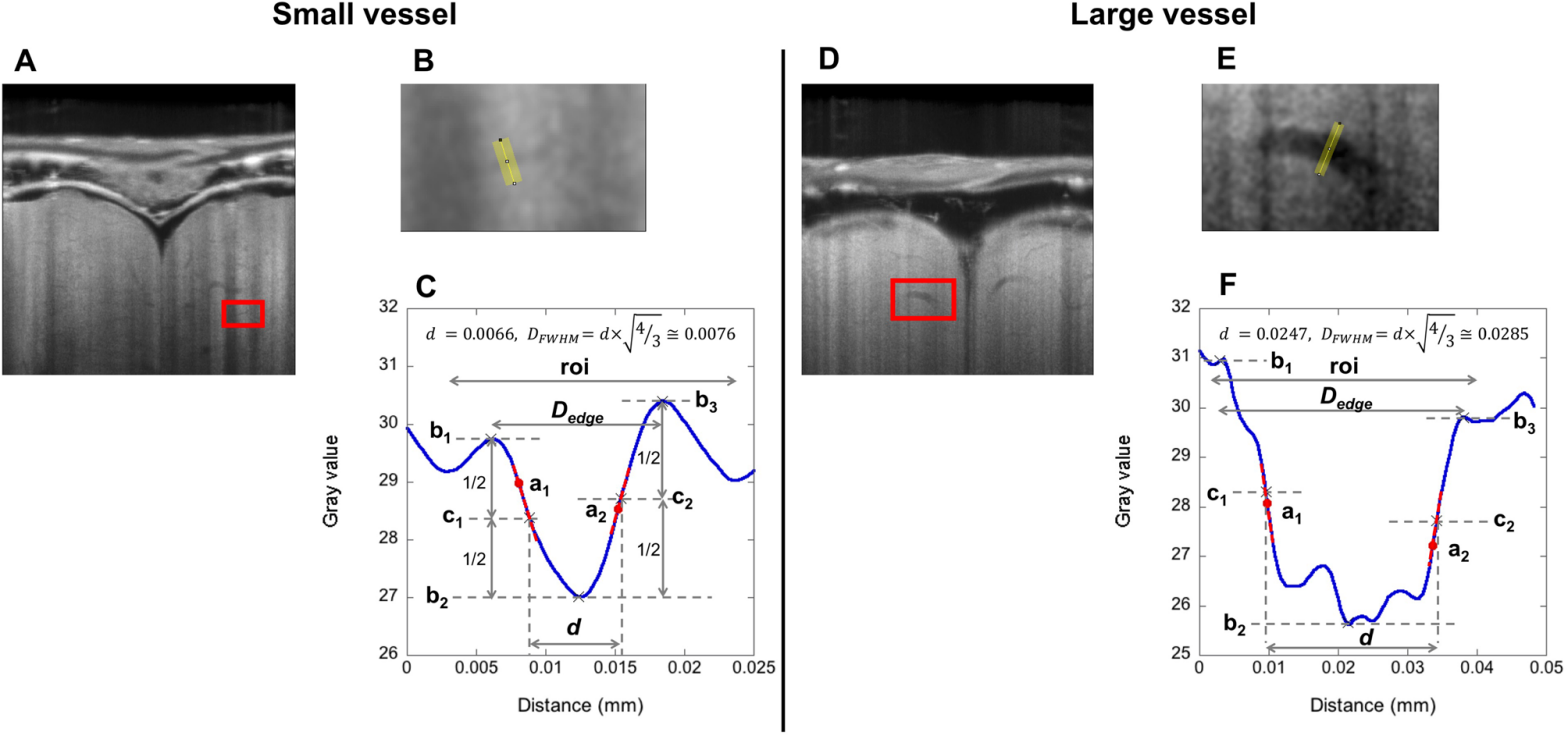
Diameter measurements of cerebral blood vessels in medaka telencephalon. (**A**,**D**) Axial OCT image containing a small (**A**)/large (**D**) vessel in the red rectangle (ImageJ 1.52n, https://imagej.nih.gov/ij). (**B**,**E**) Enlarged views of the red rectangle in A and D. (**C**,**F**) Intensity profile along the yellow line in B and E (KaleidaGraph v4.5.3, https://www.hulinks.co.jp/software/stat_graph/kaleida). The boundary estimates (a_1_ and a_2_) are the two steepest positions in the vessel wall profile. Reference intensities (b_1_, b_2_, and b_3_) correspond to those of the brightest areas outside the blood vessel on each side and that of the darkest area within the vessel. The intensity threshold (c_1_ and c_2_) is the average of the adjacent reference intensities.

We measured the diameters of the darker thread-like structures to obtain the minimum and maximum diameters of the visualized blood vessels following the full width at half-maximum algorithm^36^. Figure 4 shows a representative set of vascular diameter measurements in Subject 1. The measured blood vessels are indicated by red rectangles in the axial sections of the OCT images (Fig. 4A,D), while the expanded yellow lines in their magnifications correspond to cross-sectional profile positions (Fig. 4B,E). In the OCT image of Subject 1, we could confirm 7.6 μm and 28.5 μm as the minimum and maximum outer diameters, respectively (*D*_*FWHM*_ in Fig. 4C,F). As the largest possible outer diameter, we calculated the distance between the maximum-intensity points at both edges of the vessel, eventually finding 12.4 μm and 35.1 μm for the minimum and maximum outer diameters, respectively (*D*_*edge*_ in Fig. 4C,F). We repeated the procedure also in other subjects (Supplementary Figs. 3–7), finding that the minimum and maximum outer diameters of the visualized blood vessels were 8.2 ± 1.8 μm and 27.4 ± 4.3 μm (mean ± SD), respectively (*D*_*FWHM*_ in Table 1); further, the largest possible outer diameters of the minimum and maximum diameters were 14.0 ± 3.7 μm and 36.0 ± 5.2 μm (mean ± SD), respectively (*D*_*edge*_ in Table 1).

**Table 1:** Measured diameters of visualized blood vessels in medaka telencephalon.

	*d* (μm)		*D*_*FWHM*_ (μm)		*D*_*edge*_ (μm)	
	Small	Large	Small	Large	Small	Large
Subject 1	6.6	24.7	7.6	28.5	12.4	35.1
Subject 2	9.4	19.7	10.9	22.8	15.6	30.9
Subject 3	6.3	25.2	7.3	29.1	13.6	39.1
Subject 6	5.7	22.5	6.6	26.0	12.1	39.0
Subject 7	9.0	19.7	10.4	22.7	21.0	28.2
Subject 8	5.6	30.5	6.5	35.3	9.0	43.4
Mean ± SD	7.1 ± 1.5	23.7 ± 3.7	8.2 ± 1.8	27.4 ± 4.3	14.0 ± 3.7	36.0 ± 5.2

*d*, full width at half maximum of the vessel intensity profile; *D*_*FWHM*_, estimated vascular outer diameter by the full width at half-maximum algorithm; *D*_*edge*_, distance between the edges of the vessel intensity profile.

### 3D visualization of the microvasculature in medaka telencephalon

**Figure 5:**
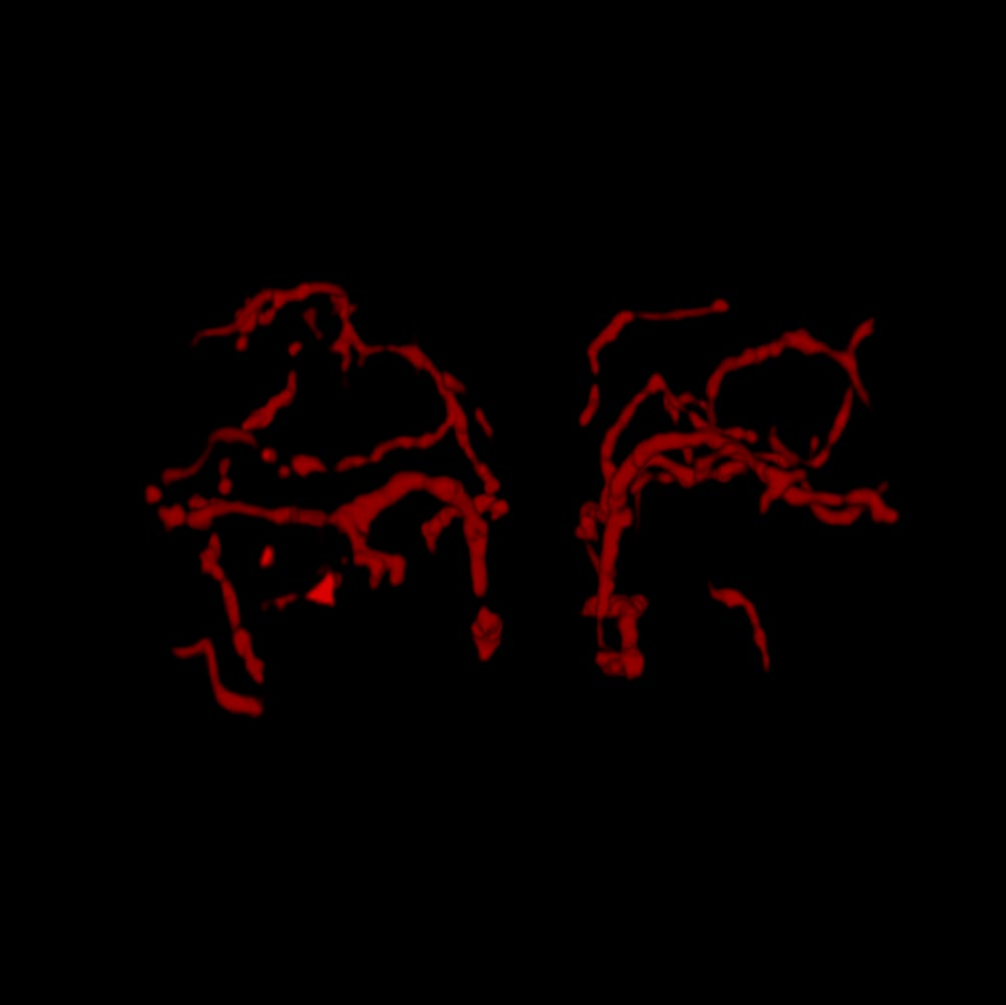
3D reconstruction of microvasculature in medaka telencephalon. Manually segmented thread-like structures in the OCT image (Subject 1 in Fig. 1) were used for the reconstruction (ImageJ 1.52n, https://imagej.nih.gov/ij).

We segmented the darker thread-like structures to visualize the 3D branching structure of the microvasculature in medaka telencephalon (Fig. 5 and Supplementary Movie 3: Media 5). Although only large structures were segmented, we succeeded in capturing the characteristics of a 3D telencephalic vascular network through volume rendering. The visualized blood vessels traveled three-dimensionally through the whole telencephalon, albeit some of them ended in its middle part. Most of the blood vessels extended rostrally on the ventral side and laterally on the dorsal side. The blood vessel network was similar and almost symmetrical between the telencephalic hemispheres.; nonetheless, we could not distinguish between arteries and veins.

## Discussion

In this study, we visualized in vivo the dark thread-like branched structures in adult medaka telencephala by using the SD-OCT following an intact approach (Fig. 1). Comparing the cross-sectional OCT images with the corresponding histological sections of the brain of the same subject, we found that blood vessels with red blood cells in the histological sections had almost the same morphological features of the dark thread-like structures in the OCT images (Figs. 2, 3). Moreover, the larger dark thread-like structures exhibited Doppler shifts in the Doppler OCT images, testifying moving particles and/or flows (Fig. 3). According to these findings, we concluded that the dark thread-like structures in the OCT images were telencephalic micro-blood vessels.

The outer diameter of the visualized telencephalic blood microvessels ranged from 8.2 to 27.4 µm (Table 1). Based on the assessment of the inner diameter of the visualized blood vessel, we could utilize the minimum full width at half maximum of the vessel intensity profile, which was measured to range from 7.1 to 23.7 µm (Fig. 4, Table 1). The minimum delineated inner diameter corresponded to 5.6 µm in Subject 8 (Table 1). To the best of our knowledge, the inner diameter of 5.6 µm is the smallest diameter that was ever visualized on in vivo OCT angiography. Based on the random distribution of red blood cells in a blood vessel that was utilized in our method, the speckle decorrelation method was used to visualize vasculature in a human retina, which reveled a spatial resolution of 18 µm × 18 µm × 5 µm^37^. Using the human retina blood flow, the Doppler OCT could detect blood vessels of approximately 120-µm diameter in vivo^38^. White et al. showed a capillary of diameter 10 µm (6 pixels × 5 pixels = 9.6 µm × 8.0 µm) in a human retina using ultra-high-speed spectral domain optical Doppler tomography^39^. With the introduction of contrast agents (gold nanoprisms), in vivo OCT was used to image the capillaries of a mouse ear, revealing a minimum diameter of < 10 µm^40^. These vessel diameters corresponded to the inner diameter and were larger than our minimum diameter, with an average of 5.6 µm (7.1 ± 1.5 µm) (Table 1). In a medaka brain, Gladys et al.^28^ showed only medaka’s morphological features in vivo, without the delineation of the cerebral microvessels.

To accomplish the intact in vivo high-resolution imaging of microvasculature, we established a special sample-manipulation procedure. The minimum outer diameter of the visualized blood vessel was about 8 μm (Fig. 4), comparable to our OCT system resolution (Manufacture guaranteed resolution: lateral, 4.0 μm; axial, 3.0 μm in air and 2.2 μm in water). In fact, the isotropic voxel sizes of our 3D OCT images were set to 1.24 µm (Subjects 1–6) and 2.06 µm (Subjects 7 and 8). However, the spatial resolution achieved in the medaka telencephalon was affected by factors, such as imperfections in the lenses or misalighnment. Owing to the delineation of telencephalic blood microvessels down to the inner diameter of 5.6 μm, we can consider that our OCT system achieved higher spatial resolution than the diameter of the visualized blood vessel. Besides, the 3D OCT imaging time was 17 min; since the high spatial resolution was particularly prone to motion artifacts, we had to reduce body movements to the level of the imaging resolution during the imaging time. By combing eugenol anesthesia and agarose gel fixing, we succeeded to restrain body movements without stopping the heartbeats of medakas. At first, we induced anesthesia up to stage 3, which caused the cessation of body and opercular movements^41^. Since the prolonged duration of stage-3 anesthesia could turn lethal^42^, we slowly switched to stage 1 of recovery, thus causing the beginning of opercular movements only^41^. After 10–20 s of stage-3 anesthesia in the eugenol solution, the medaka was transferred into a plastic holder with the agarose gel. In addition to its body fixing and moisturizing roles, the gel might prevent the medaka from fast recovering from anesthesia since bulk water is used in the recovering process and the gel may restrict the quantity of water to be used in the process. Furthermore, the recovery time for the eugenol is longer than that for the tricaine mesylate (MS-222)^43,44^. This longer recovery time might contribute to the slower recovery from stage 3 of anesthesia during the imaging time. In adjusting the optical access to the telencephalon, the XY stage and two goniometers were also used for minimizing medaka stimulation. All these allowed for keeping the medaka between stage 3 of anesthesia and stage 1 of recovery during the imaging time and visualizing microvasculatures in vivo with a high spatial resolution following an intact approach. In the hypothermia implemented by Ueno et al.^18^, the blockade of body and opercular movements associated with the maintenance of heartbeats was achieved for 50–60 min; the same anesthesia conditions were probably realized also in our OCT imaging.

The telencephalic micro-blood vessels appeared as structures darker than the surroundings in the OCT images (Fig. 1); this image contrast originated from the fluctuations in OCT signals due to intrinsic random scatterings by moving particles such as red blood cells^45,46^. Basically, OCT images are reconstructed from light reflections: when moving particles traverse the optical path, their scatterings modify the reflection of light beams, eventually causing fluctuations in the OCT signals. When we scan a brain cross-sectionally to construct a 2D OCT image, red blood cells in the blood flow scatter light beams and, if the scan rate is high enough, cause speckle changes in the image. Using the maximum A-scan rate of our OCT system (i.e., 36 kHz), we could see speckle changes in the blood vessels in a 2D tomographic image sequence of about 20 fps. Since there were no light-reflecting media other than red blood cells therein, the blood vessels appeared as dark structures with speckle changes. When we used low A-scan rate (i.e., 5.5 kHz) (low speed and high sensitivity) and averaged 20 scans, the speckle changes in the OCT signal were averaged out even in microvessels where a low flow velocity was expected; thus, visualization of the microvasculature became possible thanks to the inherent angiographic contrast. Remarkably, we did not require the injection of any fluorescence labels or exogenous contrast agents into the circulation, which might prove toxic and cause side effects such as allergic reactions^47^. Moreover, in contrast to rodent subjects, there was no need for any cranial window, thus of any surgery.

Furthermore, we found differences in shape and sizes between the histological sections and the corresponding cross-sectional OCT images (Fig. 2). The chemical fixation causes tissue shrinkage proportionally to histological characteristics; as a result, the diameter of large blood vessels that were about 20 μm in the cross-sectional OCT images shrank down to about one third in the histological sections (Fig. 2). Due to this phenomenon, smaller blood vessels might lose their lumina in a chemically fixed brain, hence the difficulty in their histological identification; therefore, the OCT images showed a more natural morphological status of the telencephalic microvasculature. Moreover, since medakas naturally swim in water, changes in the surrounding water pressure may affect the relative position of the brain. In almost all cases, the telencephalon was very close to the skull; however, one subject had a wider gap between the skull and the brain (Supplementary Fig. 6), where we observed some round shape structures. The wider gap implies a longer distance from the top of the head to the brain surface and prevents the penetration of light into the telencephalon. In addition, the structures under the skull also impeded light transmission by reflecting the irradiated imaging light on their surfaces. These phenomena led to a blurred visualization of the microvasculature in the subject with the wider skull–brain gap (Supplementary Fig. 6). Nonetheless, since medaka swims near the water surface to reach food in our aquarium, the imaging environment of our OCT system is supposed to reach a water pressure level comparable to that in a natural environment by filling the agarose gel up to near the top of the medaka head.

**Figure 6:**
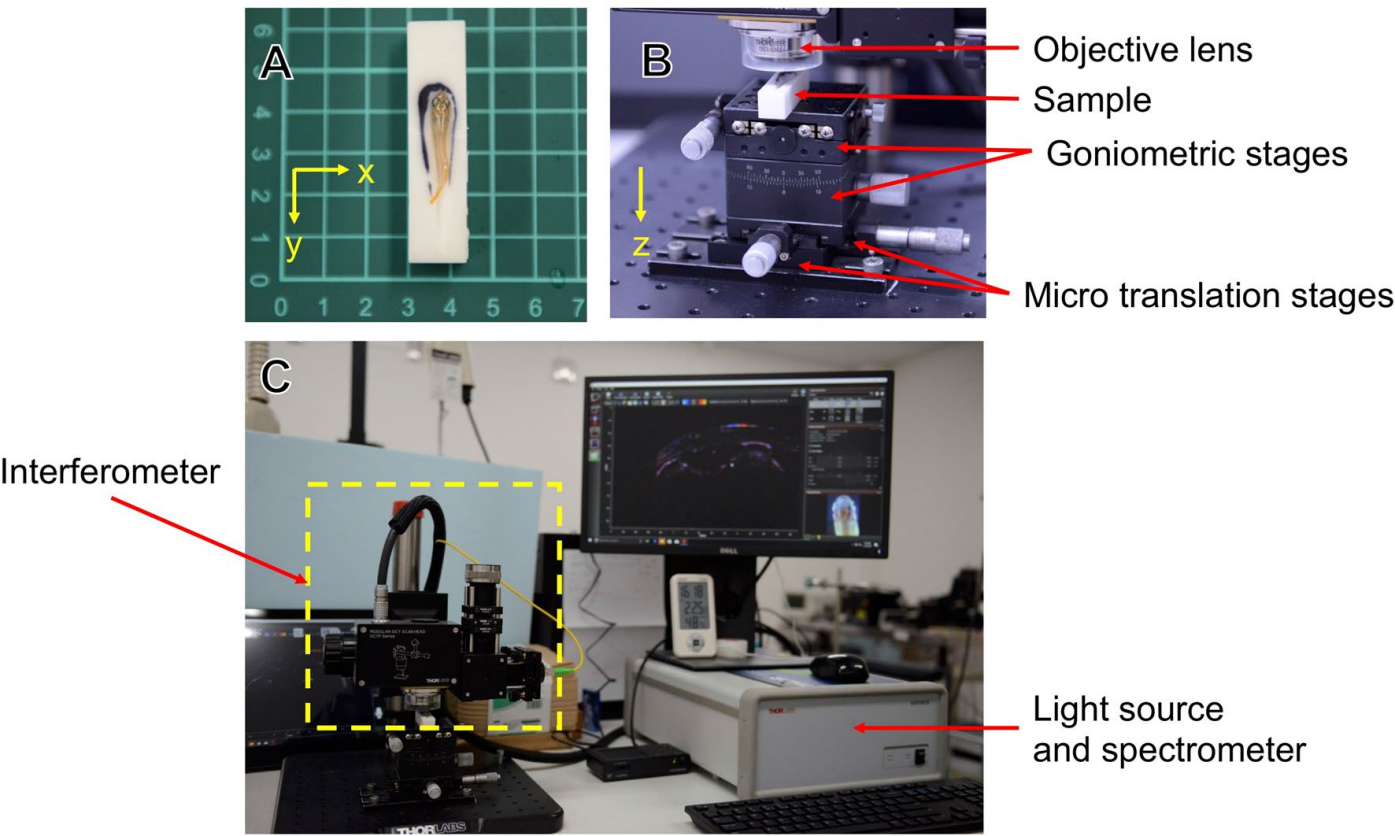
Setup of spectral-domain OCT system for investigating medaka brains. (**A**) Polyvinyl chloride sample holder. The holder has a pit where to accommodate the medaka. (**B**) Imaging stage equipped with two linear translation stages and two goniometer stages. (**C**) Spectral-domain OCT system (Ganymede II, Thorlabs). The system consists of an interferometer (left), a light source, and a spectrometer (right). The yellow arrows in A and B indicate the axis of the OCT scan coordinates.

Moreover, we visualized the blood flow in a large blood vessel in the medaka telencephalon using Doppler OCT (Fig. 3). Two methods, that is, Doppler OCT and speckle variance, have been developed in OCT angiography: the Doppler OCT is based on measuring phase shifts induced by moving scatterings^48–50^, whereas the speckle variance identifies microvasculature by calculating the interframe intensity variance of structural images, where contrast is based on different time-varying properties of blood versus solid tissue components^51^. OCT angiography based on Doppler OCT^52^ and speckle variance^53^ is widely used to characterize the cerebrovascular network of rodent brains. In this study, we tried to detect the cerebral blood flow of medaka using both methods; nevertheless, the two techniques could not detect blood flow in all the small blood vessels in both telencephalic hemispheres. This was due to the impossibility of removing noise from the visualized small vessels whose sizes were slightly larger or comparable to the spatial resolution of our OCT. Besides, the flow speed in the small vessels would be too slow for both methods and, since many small vessels run horizontally on the dorsal side of the telencephalon (Fig. 1), the current optical access from the top of the head would further hamper the Doppler OCT method. Even the large blood vessel showing Doppler shifts might not have enough flow speed for the speckle variance technique.

In the visualized microvasculature, the smaller blood vessels were distributed mainly on the dorsal side and run parallelly to the dorsal surface of the telencephalon, whereas the larger blood vessels were found in a deeper region and seemed to connect to dorsal and lateral regions (Fig. 1). These characteristics were also confirmed by SEM imaging of corrosive resin cast blood vessels of a medaka brain^54^. Since the minimum size of the leucocytes in medaka is about 6 μm^55^, which is comparable to the minimum outer diameter of the visualized smaller blood vessel, 8 μm (Fig. 4, Table 1), we could come close to visualize the smallest blood vessels in the telencephalon of medaka. Further, we measured the major and minor axes of red blood cells in the histological sections (Fig. 2), which were 7.4 ± 0.9 μm and 3.0 ± 0.6 μm (mean ± SD), respectively (Table 2); in addition, the minimum full width at half maximum of the vessel intensity profile of 7.1 ± 1.5 μm (mean ± SD) in Table 1, which we considered as an inner diameter of a visualized blood vessel, would be large enough to accommodate blood cells. These measurements also support the aforementioned arguments.

**Table 2:** Measured major and minor axes of red blood cells in histological sections.

RBC	Slice 1		Slice 2L		Slice 2R		Slice 3L		Slice 3R		Slice 4	
	*a*	*b*	*a*	*b*	*a*	*b*	*a*	*b*	*a*	*b*	*a*	*b*
1	7.3	2.8	5.9	2.3	7.6	1.9	7.8	3.3	5.4	2.8	7.0	2.2
2	7.0	3.0	8.7	3.6	6.6	2.2	–	–	–	–	7.0	3.9
3	7.4	2.1	7.7	3.7	–	–	–	–	–	–	6.5	3.8
4	7.5	3.3	8.1	3.5	–	–	–	–	–	–	6.9	2.6
5	–	–	8.3	3.0	–	–	–	–	–	–	6.1	3.7
6	–	–	7.1	2.9	–	–	–	–	–	–	–	–
7	–	–	8.3	3.0	–	–	–	–	–	–	–	–
8	–	–	8.4	3.2	–	–	–	–	–	–	–	–
9	–	–	9.0	3.9	–	–	–	–	–	–	–	–
Mean ± SD	***a***, 7.4 ± 0.9; ***b***, 3.0 ± 0.6											

RBC, red blood cell; Slice, histological slice in Fig. 2; L (R), blood vessel on the left (right) side; *a*, major axis in μm; *b*, minor axis in μm.

Moreover, we extracted the 3D structures of the major blood vessels from the 3D OCT images and reconstructed a 3D angiographic model (Fig. 5). We found similar and almost symmetrical structural and directional patterns between the two hemispheres, as observable in the SEM images by Isogai et al.^54^. Although inter-hemisphere differences in the major blood vessels were relatively small, we found great intersubject differences in the architecture and connectivity of the telencephalic blood vessels, especially the smaller ones; besides, the inter-hemisphere differences of the smaller blood vessels were greater than those of the larger ones. These findings indicated the importance of time-series analyses of individual medakas, which was made possible by the present study.

The high penetration depth, spatial resolution, and A-scan rate of our OCT system, along with the sample-manipulation procedure, realized intact in vivo imaging and visualization of the telencephalic microvasculature in adult medakas without any strictly angiographic support. This study showed possibilities that intact in vivo imaging could be performed freely for different stages of the same fish subject to evaluate cerebrovascular alterations, although quantitative measurement of blood velocity and artery–vein discrimination could not be performed. Nevertheless, not only diameters, architecture, and connectivity, but also microvascular density and tortuosity could be obtained using the 3D angiographic image. However, due to the limited spatial resolution, imaging depth and area, and signal sensitivity, especially in deeper regions, the intact in vivo imaging in this study could not delineate the whole telencephalic vasculature. Thus, detailed analysis of the 3D angiographic image should focus on a specific telencephalic region. Future work should focus on determining the percentage of the telencephalic vasculature that can be visualized in this study, comparing the in vivo OCT images with a ground truth such as SEM images^54^. So far, in vivo imaging studies of small fish are limited mainly to the early stage where body transparency remains; in fact, only a few imaging techniques for adult small fish exist, especially for those beyond one year post-hatching due to the age-related loss of body transparency. Ueno et al.^18^ non-invasively evaluated the hepatic steatosis level of NAFLD model in adult medakas using MR microscopy, following each disease progression pattern. This modality can also non-invasively access the brain of medaka; however, its spatial resolution is not sufficient to visualize the cerebral blood vessels. By contrast, the high-magnification lens of our OCT system gives a spatial resolution high enough to visualize microvessels; nonetheless, the field of view becomes narrower. Adult medakas, whose body size is smaller than that of zebrafish, are thus preferable for intact in vivo OCT angiography. Concerning penetration depth, the small brain size also facilitates the visualization of deeper vasculature; in addition, the adult medaka body skin remains slightly more transparent than other animal models, and its transparency is also suitable for OCT. In rodents, an optical window has to be achieved by invasive surgery to perform in vivo OCT imaging. In animal models for metabolic syndrome and aging, pathological conditions causing cerebrovascular changes were reported, especially at microscopic levels^56–58^. In addition to rodent models, medaka and zebrafish models for metabolic-syndrome-related diseases have been developed^11–13,18,59–61^. With the intact in vivo OCT angiography, it will be possible to follow up on the progression of metabolic-syndrome-related cerebral disorders in individual medaka models. In fact, the tortuosity of a cerebral blood vessel is an indictor of metabolic syndrome-related disorders. In this study, even a larger basal telencephalic vessel showed large intersubject variations in the quantified tortuosity (Supplementary Figs. 7–10; mean, 1.20; SD, 0.145; skewness, 0.473; kurtosis, − 1.48). However, in a smaller telencephalic vessel, errors and interobserver variations in the manual segmentation may influence the quantitative results more than that in the larger vessels. The accuracy in the manual segmentation should be addressed in a future study. Since the telencephalic microvasculature shows large intersubject variations, this method may provide new insights into the influence of pathological conditions on cerebral blood vessels. Moreover, behavioral alterations due to pathological changes may be investigated through intact in vivo OCT angiography using the same individual medaka disease models.

**Figure 7:**
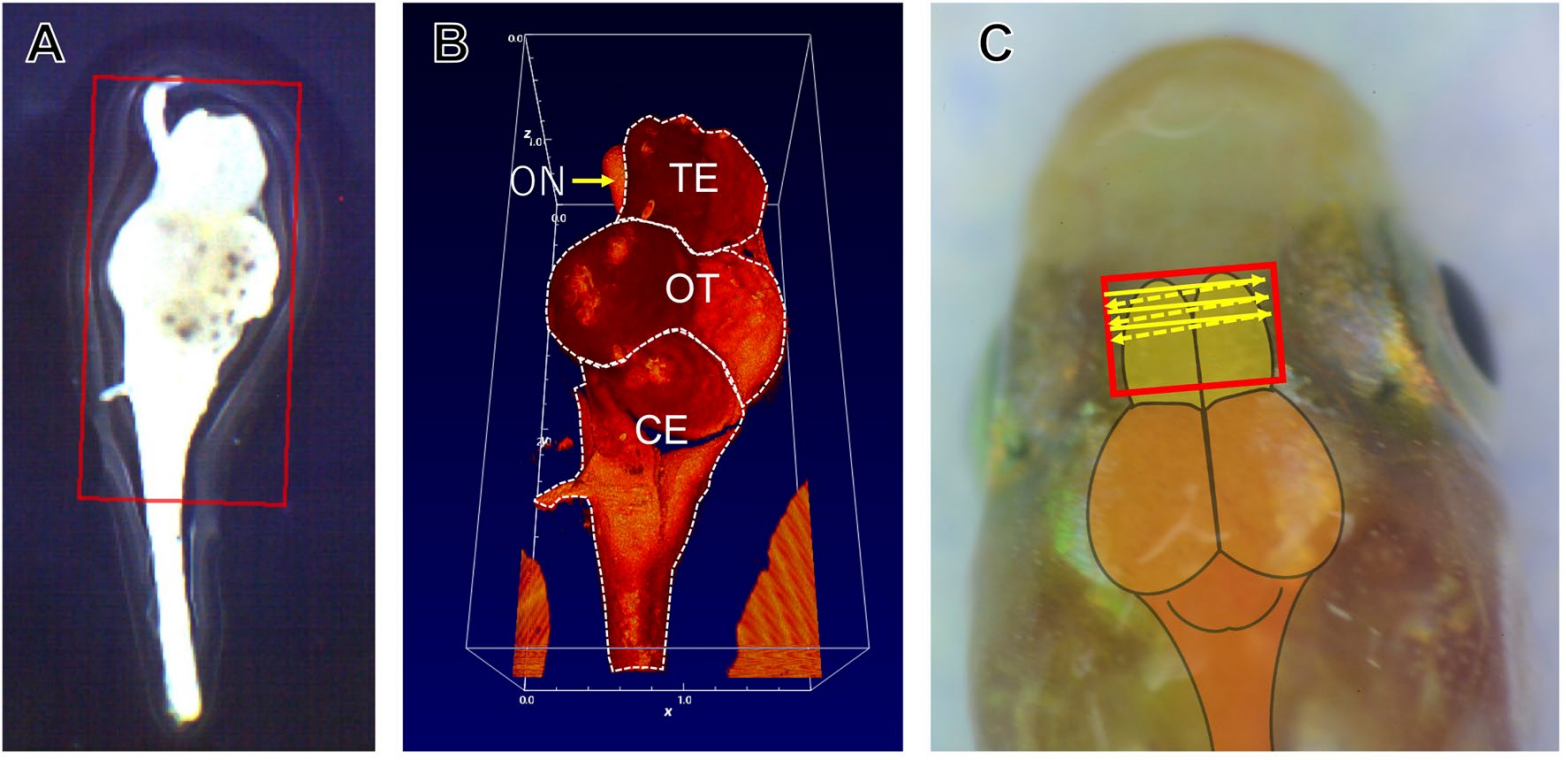
Brain of adult medaka. (**A**) Extracted adult medaka brain. (**B**) 3D-rendered SD-OCT image of the medaka brain in the red rectangle (1.92 × 3.96 mm) in (A) (ThorImage®OCT v4.4, https://www.thorlabs.com/newgrouppage9.cfm?objectgroup_id=7982). The white dashed lines indicate medaka brain regions. (**C**) Relative position of the brain in the medaka head (ThorImage®OCT v4.4, https://www.thorlabs.com/newgrouppage9.cfm?objectgroup_id=7982; Procreate® v4.3, https://procreate.art). The approximate scan area and directions are shown as the red rectangle (x, 1.50 mm; y, 0.70 mm) and yellow arrows, respectively. ON: optic nerve. TE: telencephalon. OT: optic tectum. CE: cerebellum.

In conclusion, we successfully performed intact in vivo OCT imaging and showed the 3D distribution of the telencephalic microvasculature in adult medakas. The combination of high penetration depth and high resolution within the OCT system using adult medakas made this achievement possible. The minimum outer diameter of the visualized blood vessels was 8 μm, which is comparable to the minimum leucocyte size in medaka, that is, 6 μm. The 3D-visualized microvasculature showed substantial intersubject variations; thus, follow-up studies of individual medaka disease models are crucial to appropriately take intersubject variations into account. Monitoring of individual cerebrovascular changes through intact in vivo OCT imaging could play an important role in studies on the cerebral complications of metabolic syndrome, including stroke and cognitive impairment.

## Methods

### Ethic statement

All procedures were in accordance with the Japanese national guidelines and approved by the Animal Experimentation Committee of Kyoto University (Permit Numbers: Med Kyo 17,028–2, 18,002, 19,007).

### Maintenance of medaka

The adult medakas were maintained in a water-recirculating aquarium at 26 ± 1 °C with a photoperiod of 14 h of light and 10 h of darkness. Medakas were fed with fish food (Otohime B1, Marubeni-Nisshin Feed, Co., Ltd., Tokyo, Japan) twice a day on weekdays, once a day during weekends.

### Sample preparation and manipulation

We used seven adult medakas of the wild-type (i.e., the Kyoto-cab strain, a sub-strain of Cab), including three males and four females of about one year of age.

At first, each medaka was anesthetized using 0.02% eugenol (4-allyl-2-methoxyphenol) solution (Eugenol, Nacalai Tesque, Inc., Kyoto, Japan) for 10–20 s. Then, the anesthetized medaka was placed in a prone position in a hollow of a polyvinyl chloride holder (Fig. 6A). The hollow was filled with agarose gel (Agar, powder, Nacalai Tesque, Inc., Kyoto, Japan) beforehand to fill the small gaps between the holder and the medaka. After placing the medaka, further agarose gel was added to reduce the movements, prevent drying, and prolong the anesthetic effect during the imaging time. Then, we put the holder containing the anesthetized medaka on an imaging section consisted of a XY positioning stage combined with a two-axis goniometer stage (Fig. 6B), which enabled position adjustments without touching the sample. After OCT imaging, the medaka was returned back to the aquarium.

### Optical coherence tomography

To visualize the telencephalic microvasculature of medakas in vivo, we used a commercially available SD-OCT^40,62–64^ system (Ganymede II, Thorlabs, Inc., Newton, NJ) (Fig. 6C) equipped with a 10 × lens with an effective focal length of 18 mm and a working distance of 7.5 mm (LSM02-BB, Thorlabs, Inc., Newton, NJ). The light source of our OCT system was a superluminescent diode emitting broadband low-coherent light centered at 900 nm.

In an SD-OCT system, the axial resolution limit and maximum imaging depth are determined using the bandwidth and the sensor number of a spectrometer^40^. In our spectrometer, the bandwidth was 800–1018 nm and the number of sensors was 2048. Therefore, the maximum imaging depth in air changed to 1.91 mm. We only considered the absolute values of the Fourier-transformed spectral data; the axial resolution limit was 1.91 mm / (2048/2) = 1.865 μm. Owing to finite data sampling, we applied a tapered cosine window of 0.4 to the spectral data, and; the axial resolution limit in air changed to 2.84 μm. In cases of water and tissue, refractive indices *n* changed the resolution limit to 2.14 μm (*n* = 1.33) from 2.06 to 2.10 μm (*n* = 1.38 and 1.35), respectively.

In an OCT system, the lateral resolution limit is determined based on the 1/e^2^ spot size of a probing beam using an equipped scan lens^64^. Assuming the beam diameter before the lens to be 4 mm, the spot sizes were calculated as the functions of the scan angle (± 7.5°) for wavelengths of 900 and 930 nm, and the 1/e^2^ beam diameters at the field of focus were 3.83 and 3.96 µm, respectively. Owing to the spectral bandwidth, in our system, the lateral resolution limit was set at 4.0 μm.

Using a caliblation target, lens aberrations were corrected, and the positions of Galvano mirrors were adjusted. The position of a pixel in a monitor video camera image was also calibrated in a sample area. The pixel sizes in the lateral directions (x and y) could be determined arbitrarily in a rectangular scan area on the monitor video camera image. The pixel spacing along the axial direction was determined to be 1.865 μm using the spectrometer.

A depth scan rate of the OCT (A-scan rate) was set to 36 kHz in image adjustment and Doppler imaging, and 5.5 kHz in 3D imaging. The system was controlled by a provided software (ThorImage®OCT v4.4, Thorlabs, Inc., Newton, NJ), which also provided the Doppler OCT mode.

### Data acquisition

In this study, we studied the telencephalon of medakas— which is equivalent to the cerebrum in humans—using OCT. Figure 7A shows an extracted adult medaka brain. The major brain regions of medaka, such as the telencephalon (cerebrum), optic tectum (mid-brain), and cerebellum, are superimposed on the 3D OCT image (Fig. 7B). The telencephalon is at the tip of the brain and between the eyes (Fig. 7B,C). We searched for the center of the telencephalon using a 2D real-time OCT imaging, whose 0.5–1.0-mm scan line was set between the eyes on a live video image of the OCT system. Continuous 2D scans provide real-time axial section (x–z plane) images of the medaka brains. The center of telencephalon was adjusted by moving the medaka using the XY stage. To avoid saturation artifacts, which occurred on flat reflective surfaces and sometimes appeared in the top of the medaka head, the XY stage was tilted using two goniometers connected to the stage. After having decided the sample position, the fields of view were set to 0.95 mm × 0.70 mm × 1.91 mm (Subjects 1–6) and 1.58 mm × 0.70 mm × 1.91 mm (Subjects 7 and 8) on the head of the medaka, comprising the telencephalon. The image sizes of the 3D images were 768 × 256 × 1024 pixels (Subjects 1–6) and 768 × 358 × 1024 pixels (Subjects 7 and 8), with a data size of about 1 GB. The A-scan rate was 5.5 kHz; further, 20 depth A-scans were averaged. The directions of the raster scan are shown in Fig. 7C. The total scan time of the 3D image was about 17 min.

### Image analysis

Saved raw data of 3D OCT images were imported using ImageJ^65^ and resaved as an image stack in TIFF format. The voxel size of saved data was anisotropic. To correct the aspect ratio of the OCT images, we resized the data from 768 × 256 × 1024 to 768 × 566 × 1544 pixels and set the interval of each x–y–z slice as 1.24 μm (Subjects 1–6). We also resized the data from 768 × 358 × 1024 to 768 × 340 × 929 pixels and set the isotropic voxel size at 2.06 μm (Subjects 7 and 8). To enhance the visibility of the microvessels in a cross-sectional image, we applied a Gaussian filter (σ = 15) to the perpendicular direction of a showing plane.

The diameters of blood vessels in the OCT images were measured using ImageJ following the full width at half-maximum algorithm^36^. A schematic representation of the algorithm is illustrated in Fig. 4. In the axial and sagittal section, a slice was chosen to have the clearest view of a blood vessel. Then, a perpendicular line to the longitudinal direction of the vessel was drawn manually at a position where boundaries run parallel to each other, so that the line should cross the vessel and exceed the maximum vessel diameter on both sides. The line width across the vessel was set to 7 in ImageJ, and the resulting plot profile became the averaged intensity profile of each three data lines on both sides of the selected line. The profile data points were increased to 100 by applying a cubic spline interpolation using KaleidaGraph v4.5.3 (HULINKS Inc., Japan). In the profile, we manually defined the region of interest (ROI) for diameter determination as a region between the nearest local minimum points outside the maximum edge points at both vessel sides, except for the large diameter case of Subject 8 (Supplementary Fig. 5F). In this latter case, the steepest point was outside the maximum point on the right side; thus, this steepest point was chosen as the edge point of the ROI. Following the full width at half-maximum algorithm^36^, we automatically determined the maximum values (b_1_, b_3_ in Fig. 4C), the minimum value (b_2_ in Fig. 4C), the steepest points (a_1_, a_2_ in Fig. 4C), the half-maximum values (c_1_, c_2_ in Fig. 4C), and the half-maximum points in the manually determined ROI using an in-house MATLAB code (R2019b, MathWorks, Inc., USA), which was adapted from an ImageJ plug-in^36^. We measured the distance between the half-maximum points and multiplied the distance by the square root of 4/3 to have the vessel outer diameter (*D*_*FWHM*_ in Fig. 4C). To estimate the largest possible outer diameter, we measured the distance between the maximum points (*D*_*edge*_ in Fig. 4C).

### 3D angiography of cerebral blood vessels

The OCT images were preprocessed to reduce speckle noise before the extraction of the telencephalic vasculature of the medakas. More specifically, a 5 × 5 Wiener filter was applied to each cross-section of the 3D OCT image, which was transformed into a double-precision floating-point value beforehand. Then, outliers (i.e., more than 3 standard deviations away from the mean) along the z-axis were updated with a maximum or a minimum threshold depending on their values. Following the threshold-based update rejection, the maximum and minimum values of the image were set as 65,535 and 0, respectively, and each differential value was set to a 16-bit value proportionally. Such image preprocessing was performed using an in-house MATLAB code. We also applied 3D nonlocal means filter, that is, one of the most performing and robust denoizing approaches^66^. This process was performed with in-house software using the Graphic Processor Units (GPUs) based on Compute Unified Device Architecture. Then, we manually draw ROIs on the vessels using the wand tool on ImageJ in each image of a stack and also filled them with a color. The 3D image reconstruction of medaka cerebral blood vessels was performed using a 3D-viewer plug-in for ImageJ to three-dimensionally visualize image stacks.

### Tortuosity measurements

We used the same OCT images as those in 3D angiography except for the application of the 3D nonlocal means filter. In the sagittal section, a basal large blood vessel that ran in the head–tail direction in the right hemisphere was selected. First, we chose a sagittal image stack where the basal large blood vessel was observed and took minimum intensity projection of the stack (Supplementary Figs. 7–9: A1–3, A6–8). Second, we manually drew an ROI on the vessel in the minimum intensity projection image using selection tools from Fiji^67^ and saved the segmented ROI as an 8-bit mask image (Supplementary Figs. 7–9: B1–3, B6–8). Third, we skeletonized the segmented ROI and extracted branches using the skeletonize and analyze skeleton (2D/3D) tools (Supplementary Figs. 7–9: C1–3, C6–8). Finally, in the longest branch, we calculated the tortuosity as a ratio of the branch length to the Euclidean length.

### Histological sections of medaka brain

One of the OCT-visualized medakas was anesthetized in 0.02% eugenol and then sacrificed. The medaka was fixed for 6 h at 4 °C in Davidson’s fluid^68^, which consisted of 99.5% ethanol (Ethanol, Nacalai Tesque, Inc., Kyoto, Japan), 37% formalin solution (Formaldehyde Solution, Wako Pure Chemical Industries, Ltd., Osaka, Japan), acetic acid (Acetic Acid, Wako Pure Chemical Industries, Ltd., Osaka, Japan), and distilled water; then, the medaka was fixed in 10% formalin solution at 4 °C, which consisted of diluted 37% formalin solution with phosphate-buffered salts (PBS Tablets—Calbiochem, Merck KGaA, Darmstadt, Germany). Thereafter, serial 3-μm-thick axial brain sections were cut from the tip of the telencephalon and stained with hematoxylin and eosin. Sections were observed using a microscope (ECLIPSE 80i, Nikon, Japan) through 10 × and 40 × objective lenses and a 10 × eyepiece lens, and images were acquired using a microscope camera head (DS-Vi1, Nikon, Japan).

The size of a red blood cell was measured in a histological section through ImageJ. Major and minor axes of a red blood cell were manually drawn using the line-selection tool in ImageJ and their lengths subsequently measured.

## Supplementary information

Supplementary Information 1.

Supplementary Video 1.

Supplementary Video 2.

Supplementary Video 3.

Supplementary Video 4.

Supplementary Video 5.

Supplementary Information 2.

Supplementary Information 3.

Supplementary Information 4.

Supplementary Information 5.

Supplementary Information 6.

Supplementary Information 7.

Supplementary Information 8.

Supplementary Information 9.

Supplementary Information 10.

Supplementary Information 11.

## Data Availability

The datasets generated during and/or analyzed during the current study are available from the corresponding authors upon reasonable request.
