# Supplementary material for: Intact in vivo visualization of telencephalic microvasculature in medaka using optical coherence tomography
Source: Sci Rep. 2020 Nov 16;10:19831. doi: 10.1038/s41598-020-76468-6 (PMC7669881; doi:10.1038/s41598-020-76468-6)
Supplement: Supplementary file 1 — Supplementary Information 1. [file 41598_2020_76468_MOESM1_ESM.pdf]

Supplementary Information

Intact *in vivo* visualization of telencephalic microvasculature in medaka using optical coherence tomography

Takashi Suzuki [1,2 * ¶], Tomohiro Ueno [3 * ¶], Naoya Oishi [4], Hidenao Fukuyama [5]

[1] Center for Health Science Innovation, Osaka City University, Osaka, Japan

[2] Department of Psychiatry, Kyoto University Hospital, Kyoto, Japan

[3] Human Health Sciences, Graduate School of Medicine, Kyoto University, Kyoto, Japan

[4] Medical Innovation Center, Graduate School of Medicine, Kyoto University, Kyoto, Japan

[5] Kyoto University, Kyoto, Japan

¶ These authors contributed equally to this work.

Corresponding authors

* suzuki.takashi.7e@kyoto-u.jp (TS)

* ueno.tomohiro.2u@kyoto-u.ac.jp (TU)

Supplementary Movie Legends

Supplementary Movie 1. Animations of cross-sectional OCT image stacks.
The connectivity of blood vessels in the medaka telencephalon (Subject 1 in Fig. 1) is shown in the axial (x–z plane) (Media 1), sagittal (y–z plane) (Media 2), and coronal (x–y plane) sections (Media 3) (ImageJ 1.52n, http://imagej.nih.gov/ij). (AVI)

Supplementary Movie 2. Movie of Doppler OCT shows the steady flow in a blood vessel.
The movie corresponds to Fig. 3 (Media 4) (ImageJ 1.52n, http://imagej.nih.gov/ij). (AVI)

Supplementary Movie 3. Animation of 3D vascular distribution in adult medaka telencephalon.
The movie corresponds to Fig. 5 (Media 5) (ImageJ 1.52n, http://imagej.nih.gov/ij). (AVI)

Supplementary Figures

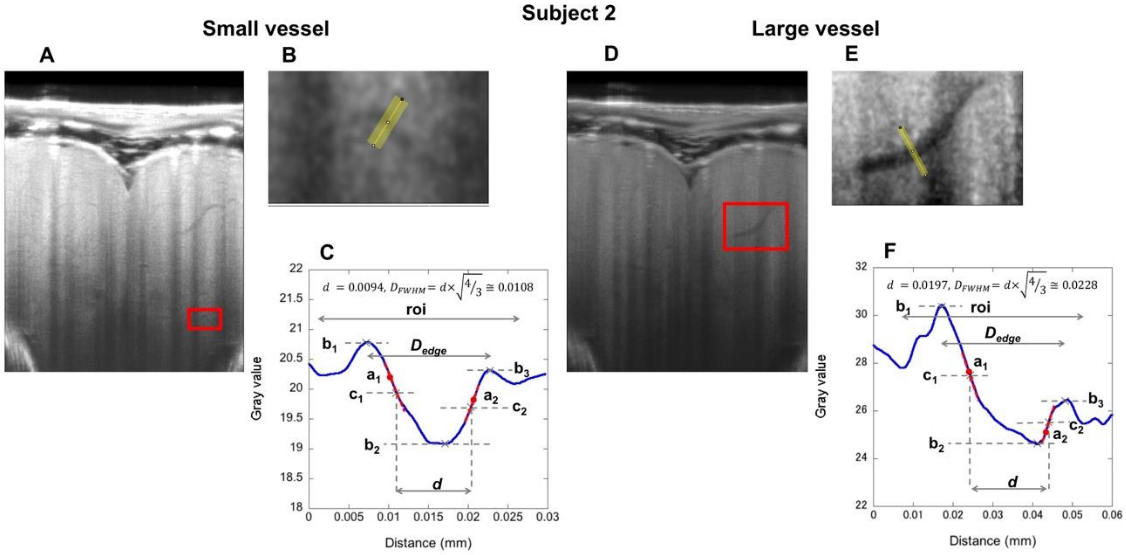

Supplementary Figure 1. Measurements of the vascular diameters in Subject 2.
Axial sections of the OCT images of the telencephalon of Subject 2 are shown in A, D (ImageJ 1. 52n, http://imagej.nih.gov/ij). The red squares indicate the studied blood vessels, and their expan ded views are shown in B, E. The diameters were extracted from the intensity profiles of the vess els along the yellow lines (C, F) (KaleidaGraph v4.5.3, https://www.hulinks.co.jp/software/stat_ graph/kaleida).

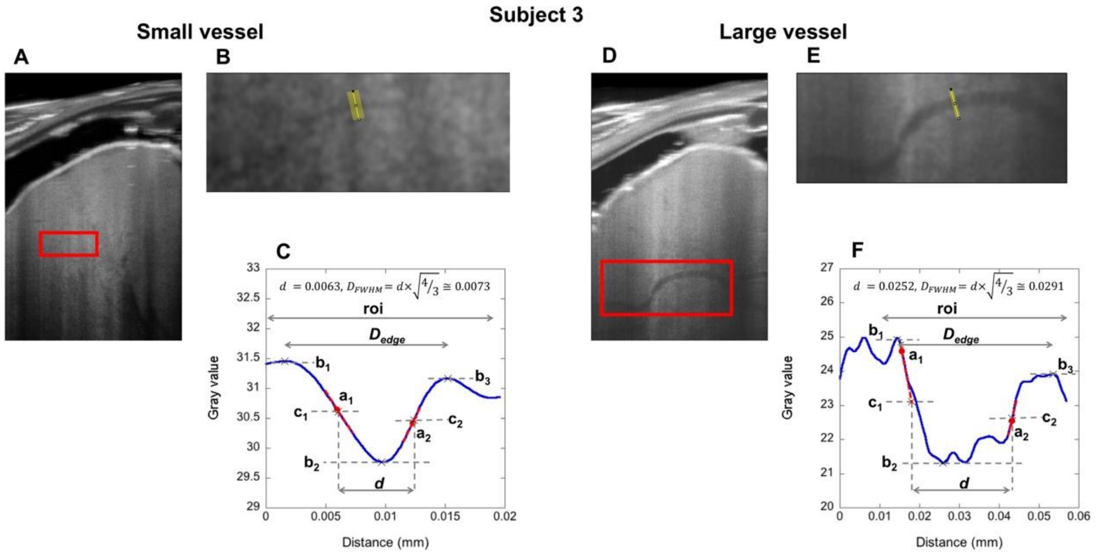

Supplementary Figure 2. Measurements of the vascular diameters in Subject 3.

Sagittal sections of the OCT images of the telencephalon of Subject 3 are shown in A, D (ImageJ 1.52n, http://imagej.nih.gov/ij). The red squares indicate the studied blood vessels and their expanded views are shown in B, E. The diameters were extracted from the intensity profiles of the vessels along the yellow lines (C, F) (KaleidaGraph v4.5.3, https://www.hulinks.co.jp/software/stat_graph/kaleida).

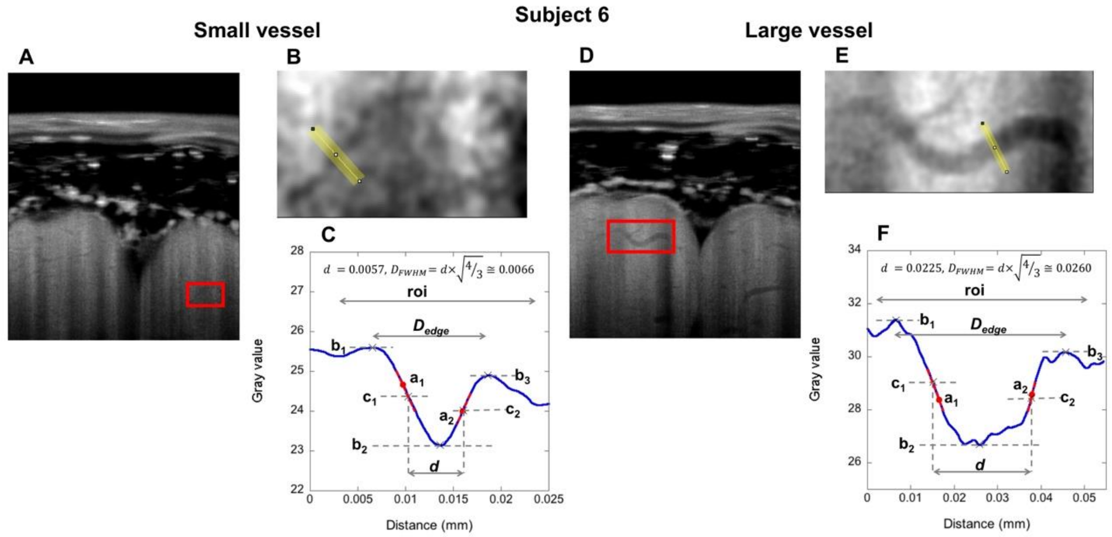

Supplementary Figure 3. Measurements of the vascular diameters in Subject 6.

Axial sections of the OCT images of the telencephalon of Subject 6 are shown in A, D (ImageJ 1. 52n, http://imagej.nih.gov/ij). The red squares indicate the studied blood vessels and their expand ed views are shown in B, E. The diameters were extracted from the intensity profiles of the vesse ls along the yellow lines (C, F) (KaleidaGraph v4.5.3, https://www.hulinks.co.jp/software/stat_gr aph/kaleida).

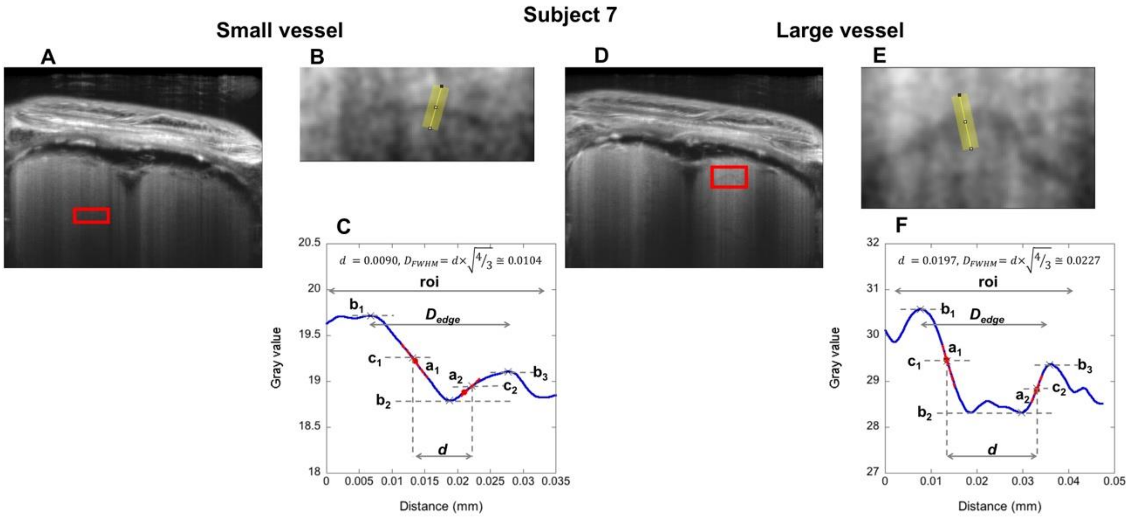

Supplementary Figure 4. Measurements of the vascular diameters in Subject 7.

Axial sections of the OCT images of the telencephalon of Subject 7 are shown in A, D (ImageJ 1.52n, http://imagej.nih.gov/ij). The red squares indicate the studied blood vessels and their expanded views are shown in B, E. The diameters were extracted from the intensity profiles of the vessels along the yellow lines (C, F) (KaleidaGraph v4.5.3, https://www.hulinks.co.jp/software/stat_graph/kaleida).

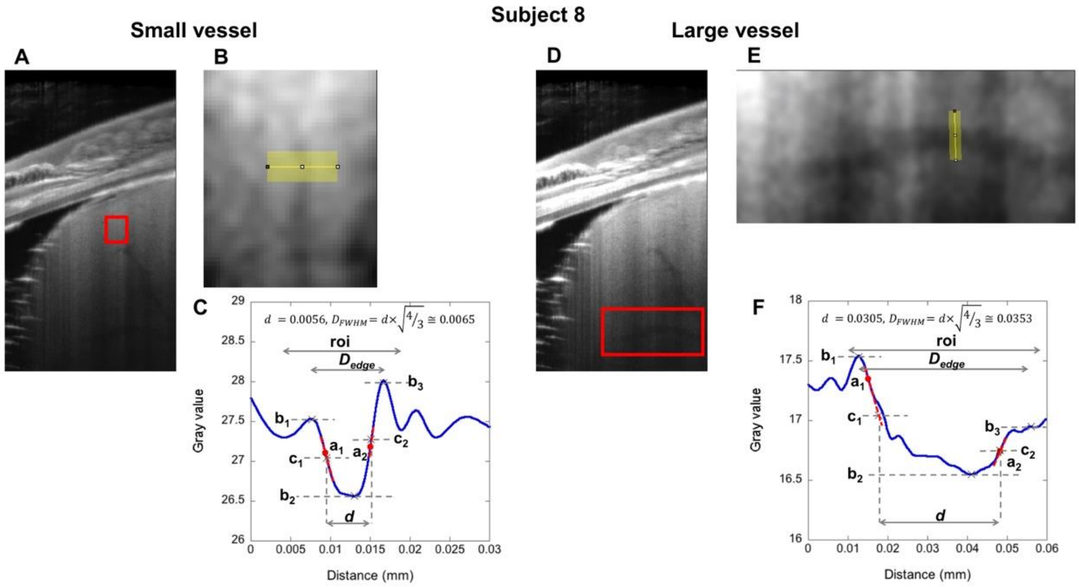

Supplementary Figure 5. Measurements of the vascular diameters in Subject 8.

Sagittal sections of the OCT images of the telencephalon of Subject 8 are shown in A, D (ImageJ 1.52n, http://imagej.nih.gov/ij). The red squares indicate the studied blood vessels and their expa nded views are shown in B, E. The diameters were extracted from the intensity profiles of the ves sels along the yellow lines (C, F) (KaleidaGraph v4.5.3, https://www.hulinks.co.jp/software/stat_ graph/kaleida).

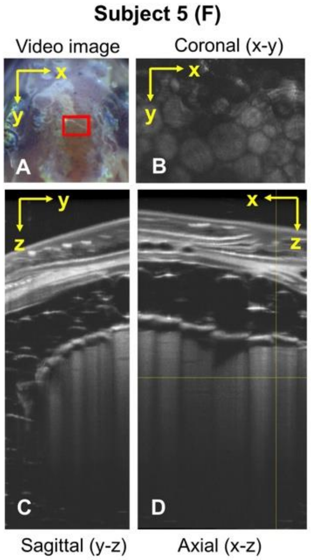

Supplementary Figure 6. Cross-sectional OCT images from another medaka.

This figure shows a representative case of a large gap between the skull and the brain surface. The gap and some structure(s) in the gap may prevent the penetration of light for imaging. In this case, it was difficult to observe the cerebral blood vessels with the OCT. (ThorImage®OCT v4.4, https://www.thorlabs.com/newgrouppage9.cfm?objectgroup_id=7982; ImageJ 1.52n, http://imagej.nih.gov/ij)

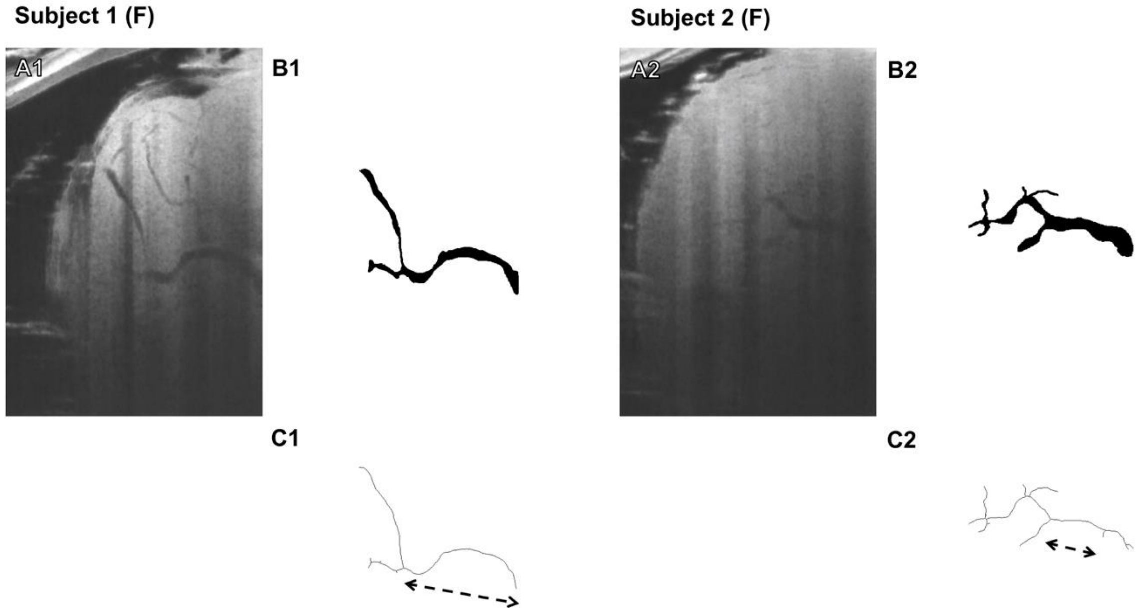

Supplementary Figure 7. Measurements of the vascular tortuosity in Subjects 1 and 2. Minimum intensity projections of sagittal stacks of the OCT images of Subjects 1 and 2 are show n in A1, A2. The sagittal stack contains a large basal telencephalic blood vessel running in the hea d–tail direction in the right hemisphere. The thickness of the stack was determined just to accom modate the head-tail direction part of the studied vessel. The segmented blood vessels are shown in B1, B2 at the same height as in A1, A2. The skeletonized blood vessels are shown in C1, C2 at the same horizontal positions as in B1, B2. The arrowed black broken lines indicate the studied l ongest branches in the skeletonized vessels. (Fiji 2.10/1.53c, https://imagej.net/Fiji)

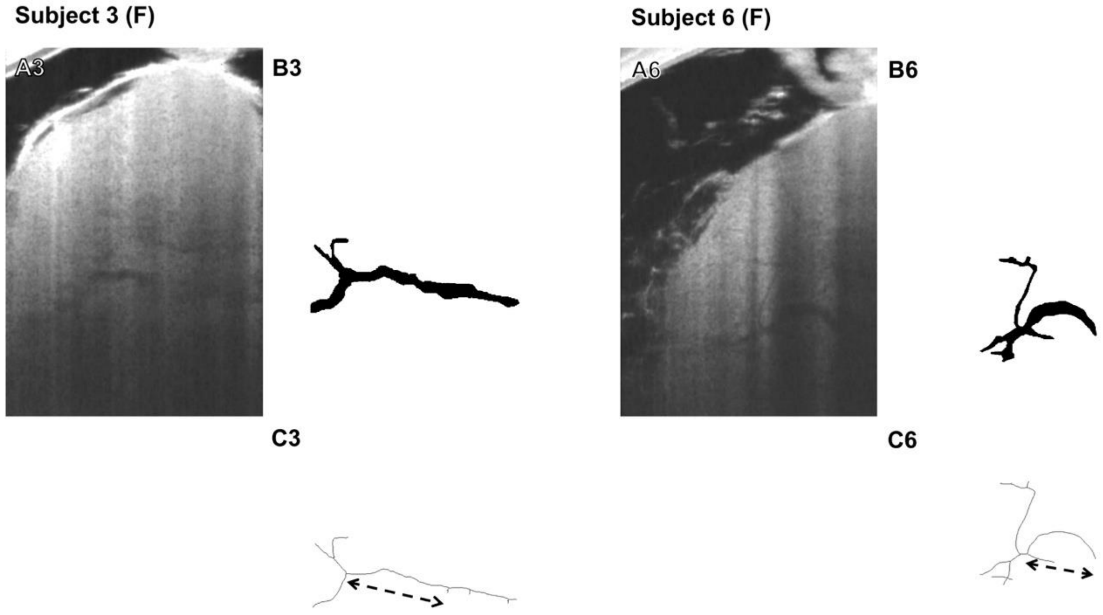

Supplementary Figure 8. Measurements of the vascular tortuosity in Subjects 3 and 6. Minimum intensity projections of sagittal stacks of the OCT images of Subjects 3 and 6 are show n in A3, A6. The sagittal stack contains a large basal telencephalic blood vessel running in the hea d–tail direction in the right hemisphere. The thickness of the stack was determined just to accom modate the head-tail direction part of the studied vessel. The segmented blood vessels are shown in B3, B6 at the same height as in A3, A6. The skeletonized blood vessels are shown in C3, C6 at the same horizontal positions as in B3, B6. The arrowed black broken lines indicate the studied l ongest branches in the skeletonized vessels. (Fiji 2.10/1.53c, https://imagej.net/Fiji)

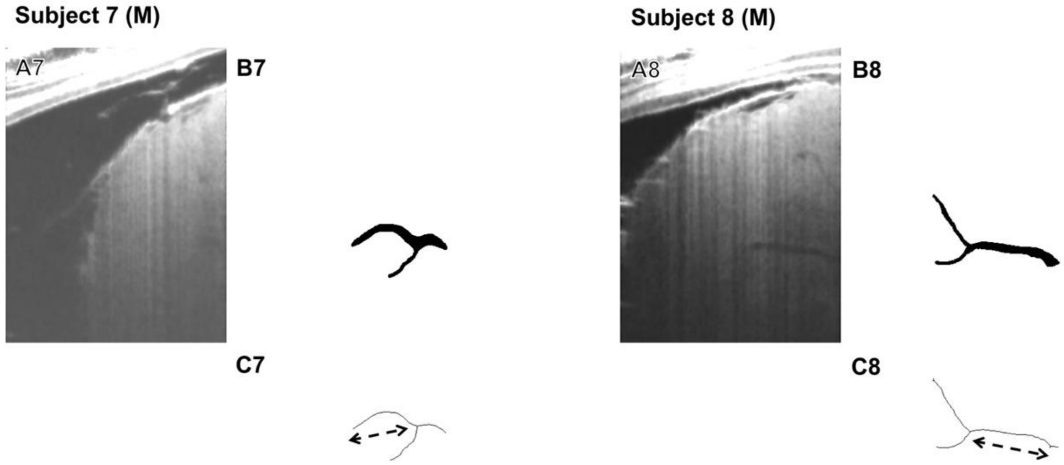

Supplementary Figure 9. Measurements of the vascular tortuosity in Subjects 7 and 8. Minimum intensity projections of sagittal stacks of the OCT images of Subjects 7 and 8 are show n in A7, A8. The sagittal stack contains a large basal telencephalic blood vessel running in the hea d–tail direction in the right hemisphere. The thickness of the stack was determined just to accom modate the head-tail direction part of the studied vessel. The segmented blood vessels are shown in B7, B8 at the same height as in A7, A8. The skeletonized blood vessels are shown in C7, C8 at the same horizontal positions as in B7, B8. The arrowed black broken lines indicate the studied l ongest branches in the skeletonized vessels. (Fiji 2.10/1.53c, https://imagej.net/Fiji)

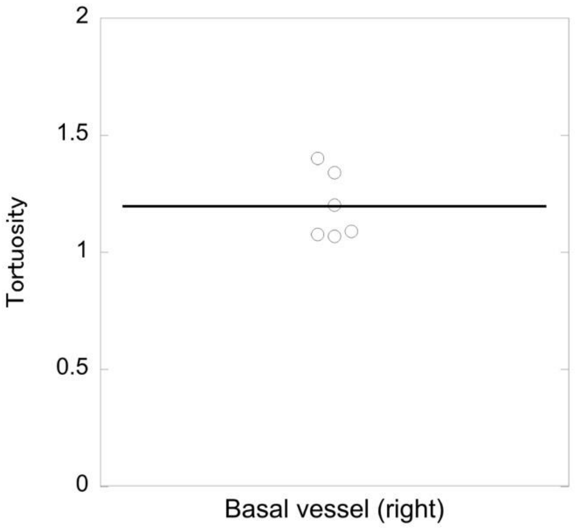

Supplementary Figure 10. Tortuosity of large basal telencephalic blood vessels in the right hemisphere.

Tortuosity of the longest branch in the skeletonized blood vessel was measured as a ratio of the b ranch length to the Euclidean length in Subjects 1–3, 6–8. The horizontal bar corresponds to the mean value. Even in large blood vessels, not small inter-subject variation appeared. Mean, 1.20; SD, 0.145; skewness, 0.473; kurtosis, −1.48. (KaleidaGraph v4.5.3, https://www.hulinks.co.jp/so ftware/stat_graph/kaleida)
