# Supplementary figures and images for: Intact in vivo visualization of telencephalic microvasculature in medaka using optical coherence tomography
Source: Sci Rep. 2020 Nov 16;10:19831. doi: 10.1038/s41598-020-76468-6 (PMC7669881; doi:10.1038/s41598-020-76468-6)

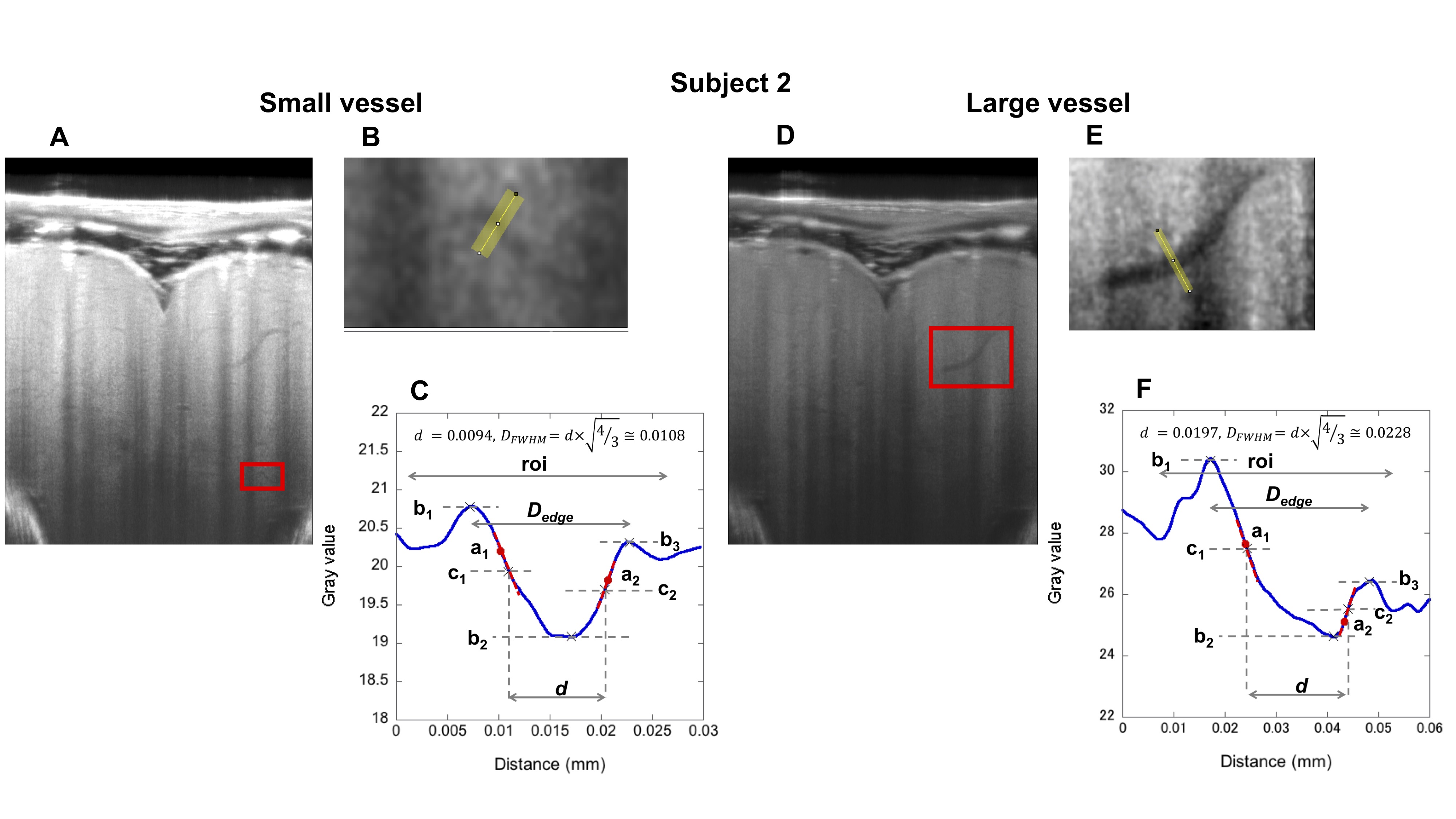

Supplement: Supplementary file 7 — Supplementary Information 2. [file 41598_2020_76468_MOESM7_ESM.jpg]

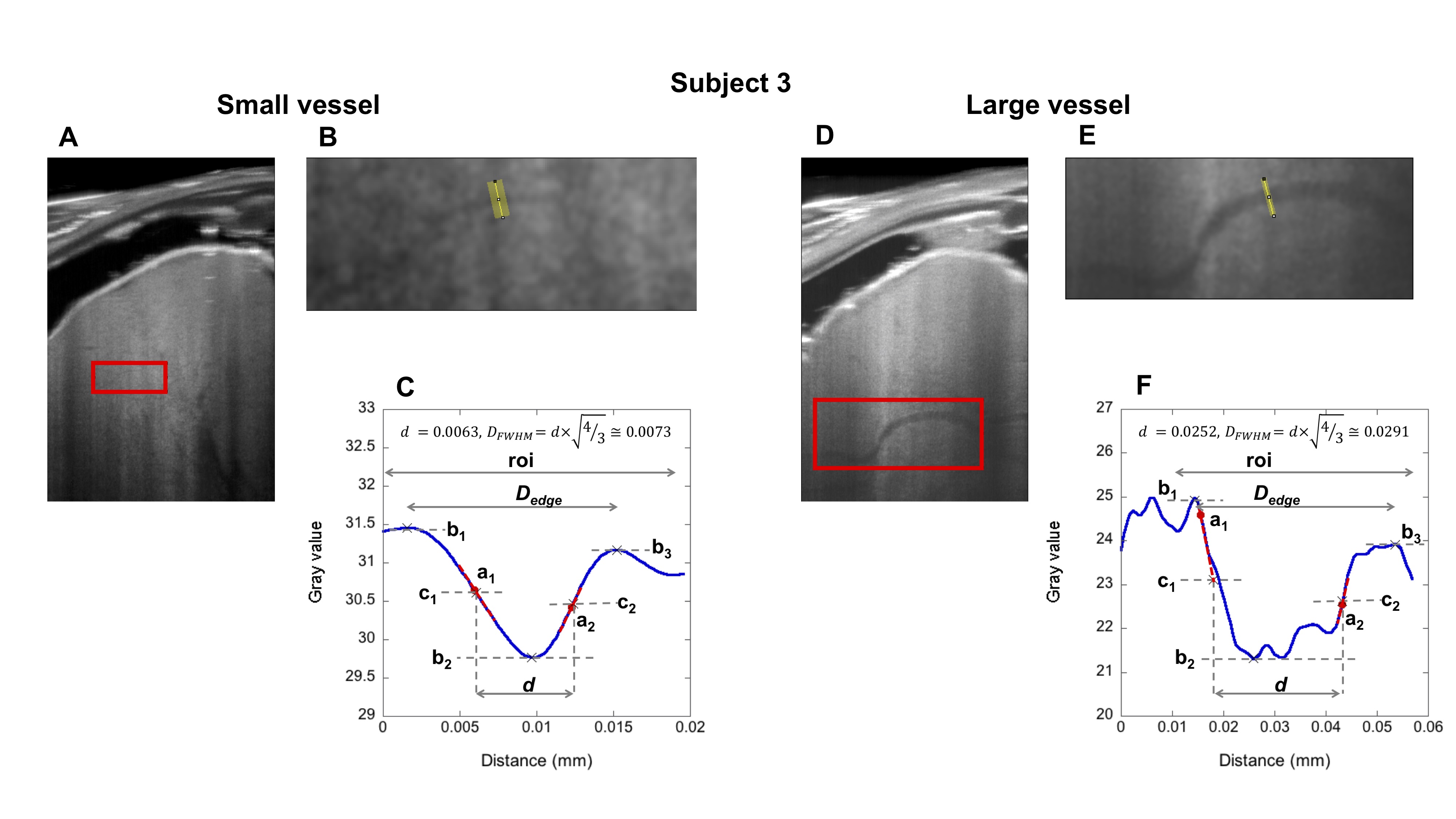

Supplement: Supplementary file 8 — Supplementary Information 3. [file 41598_2020_76468_MOESM8_ESM.jpg]

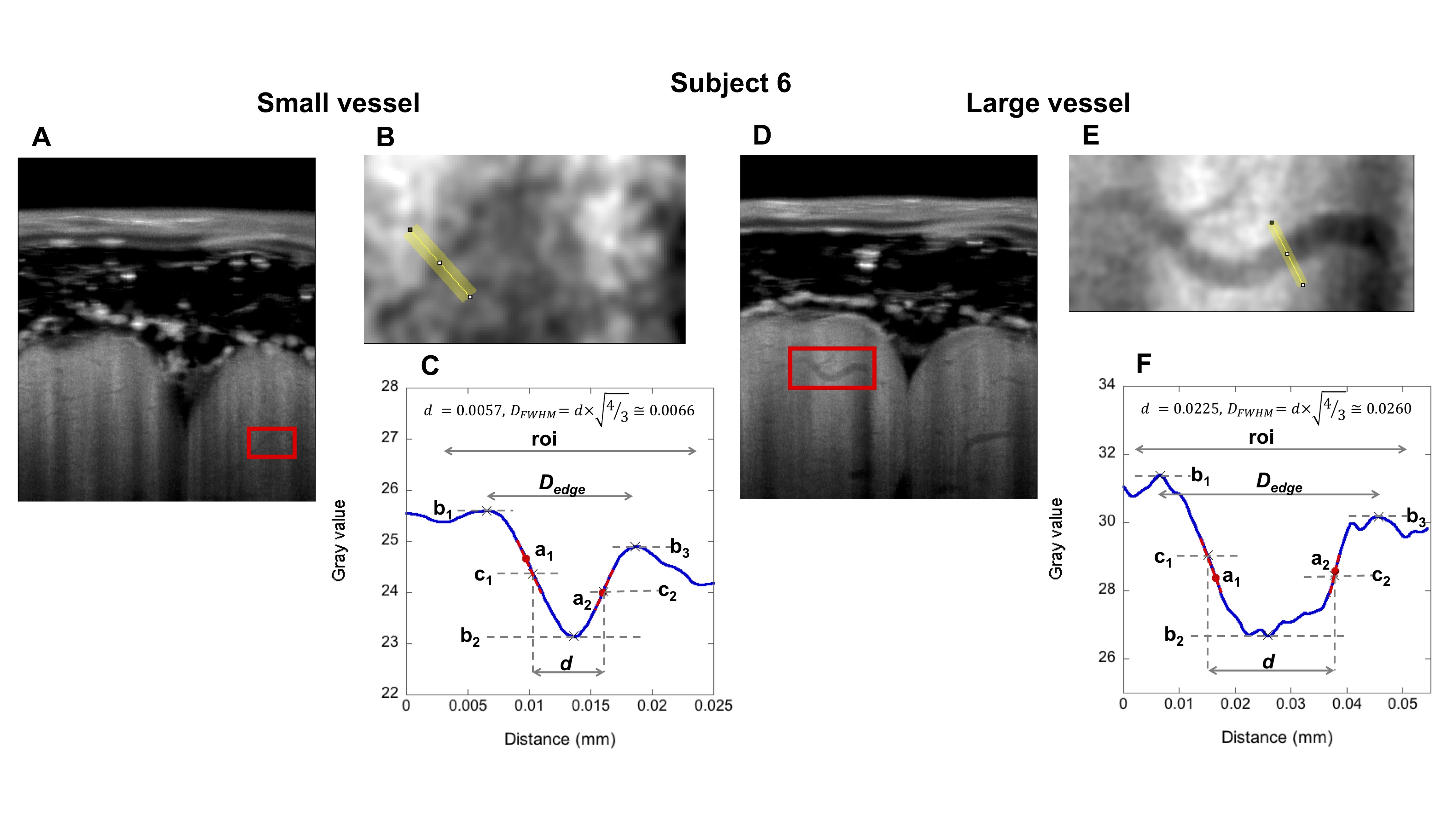

Supplement: Supplementary file 9 — Supplementary Information 4. [file 41598_2020_76468_MOESM9_ESM.jpg]

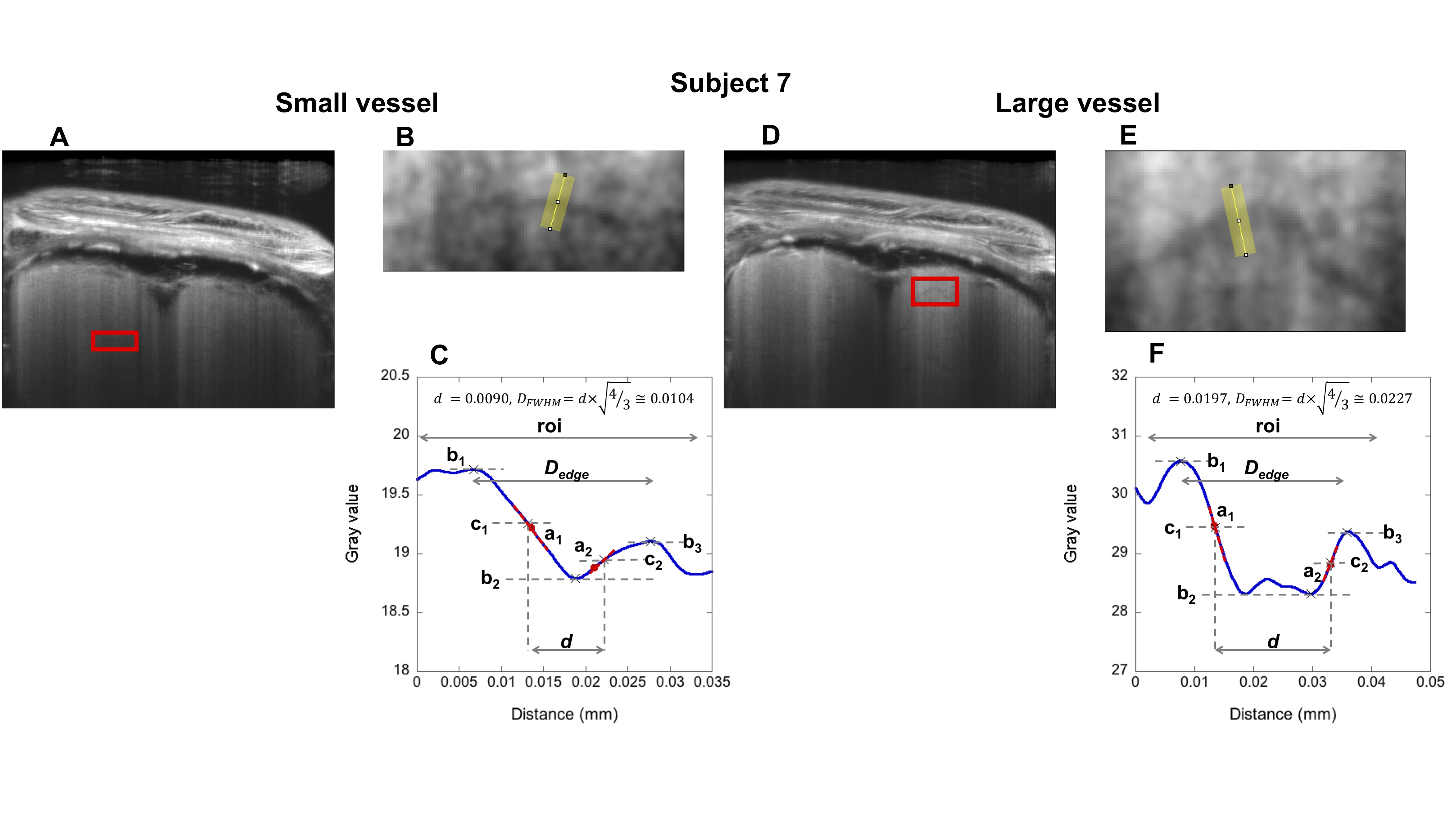

Supplement: Supplementary file 10 — Supplementary Information 5. [file 41598_2020_76468_MOESM10_ESM.jpg]

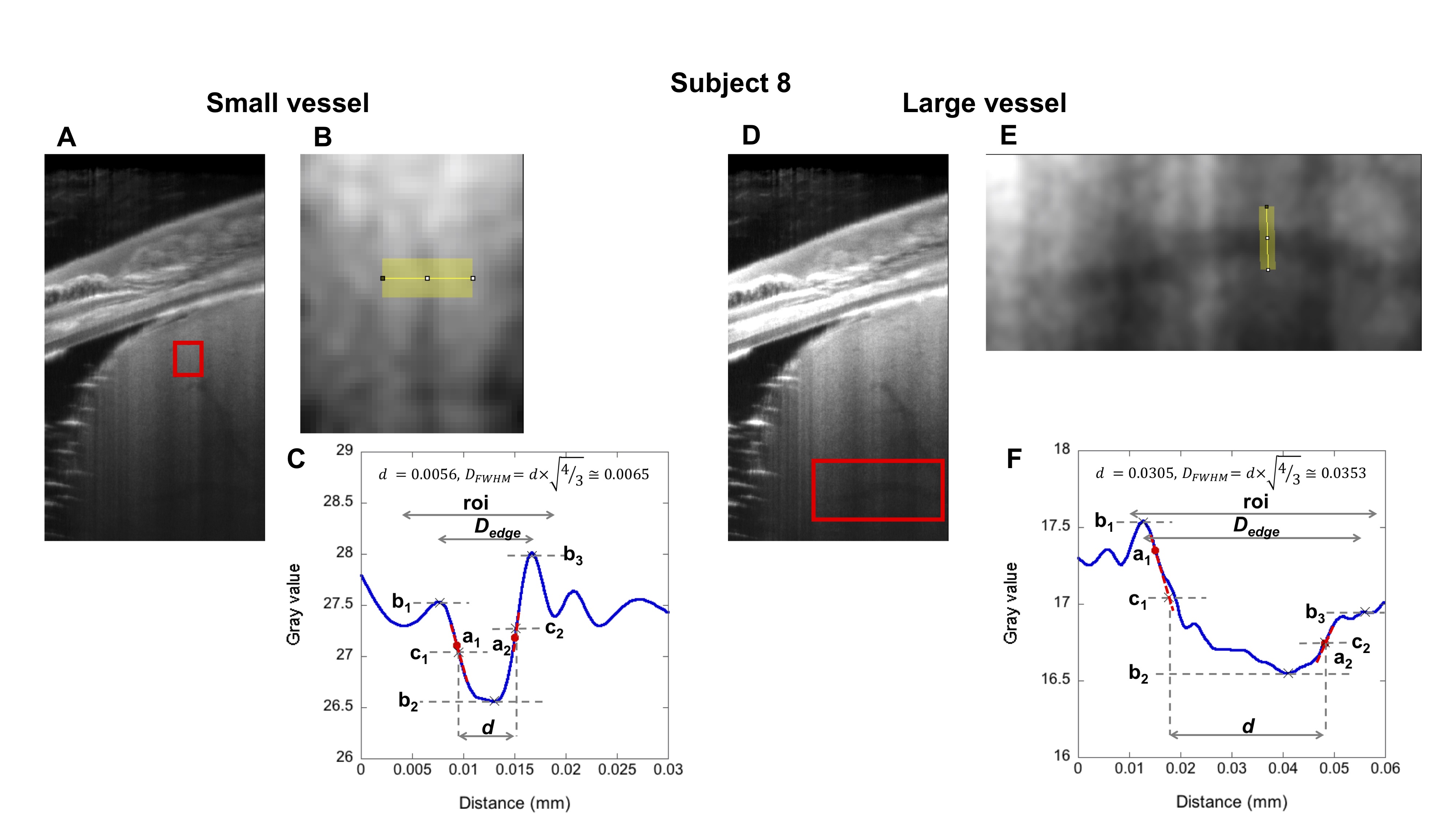

Supplement: Supplementary file 11 — Supplementary Information 6. [file 41598_2020_76468_MOESM11_ESM.jpg]

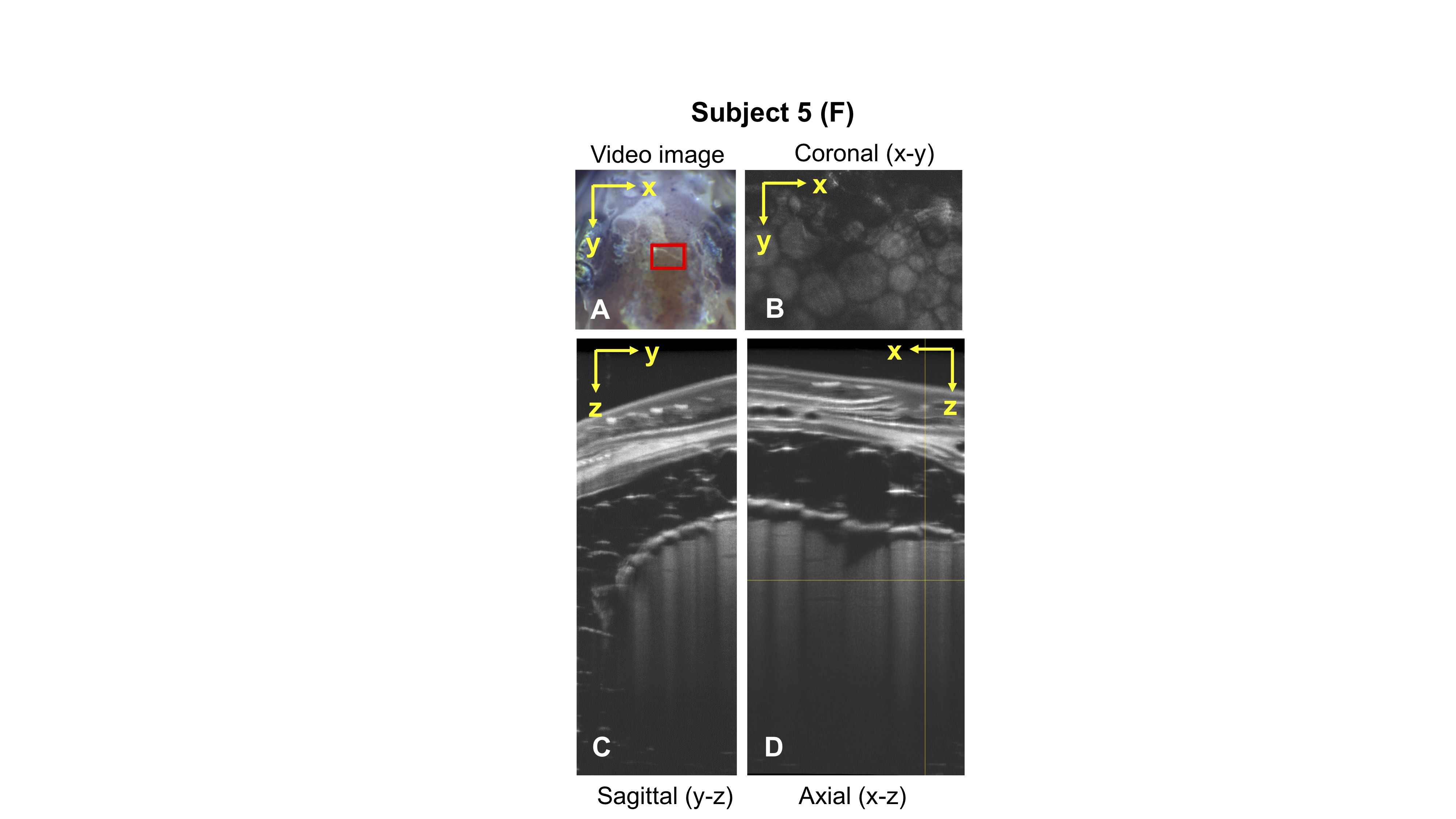

Supplement: Supplementary file 12 — Supplementary Information 7. [file 41598_2020_76468_MOESM12_ESM.jpg]

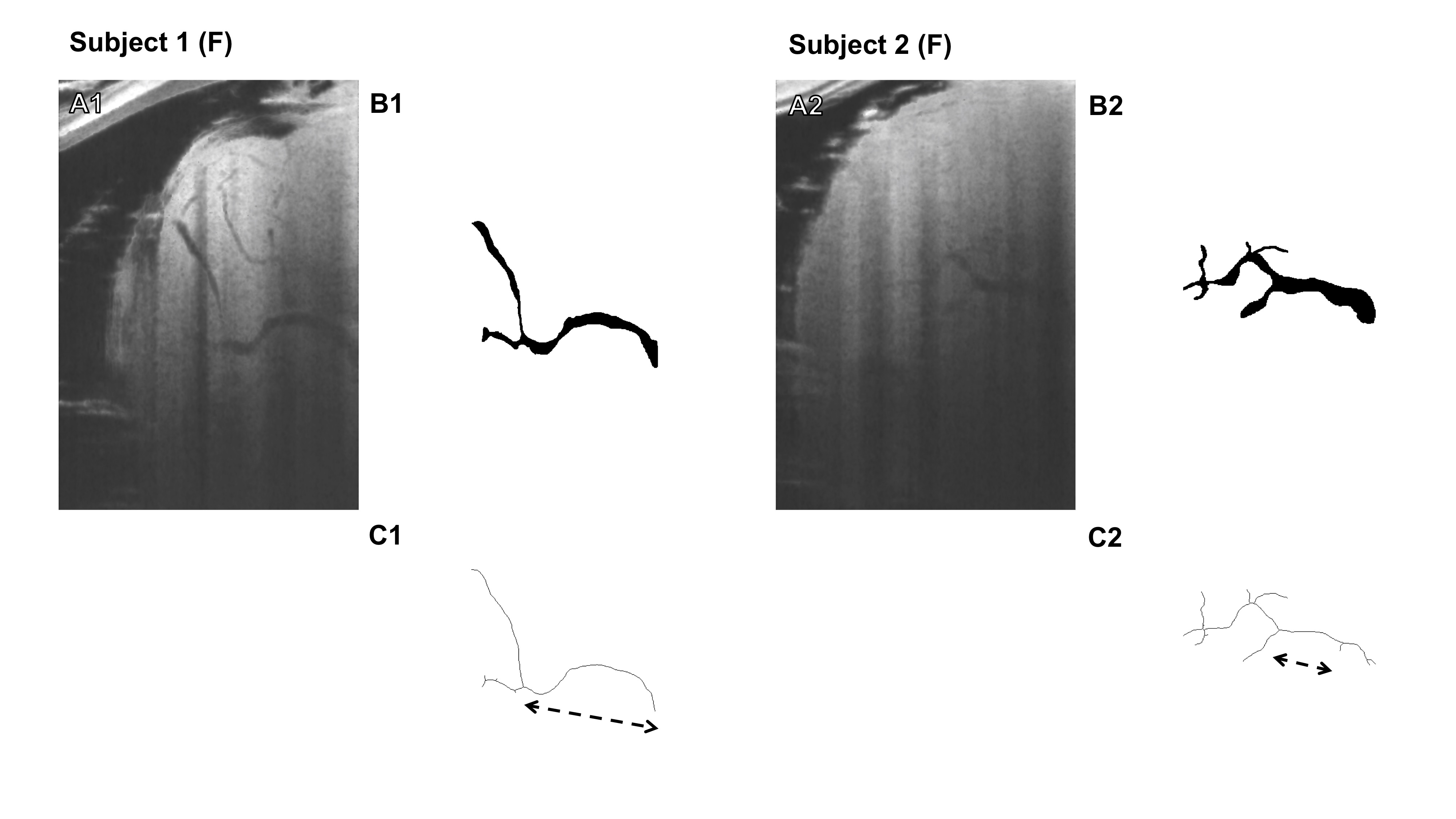

Supplement: Supplementary file 13 — Supplementary Information 8. [file 41598_2020_76468_MOESM13_ESM.jpg]

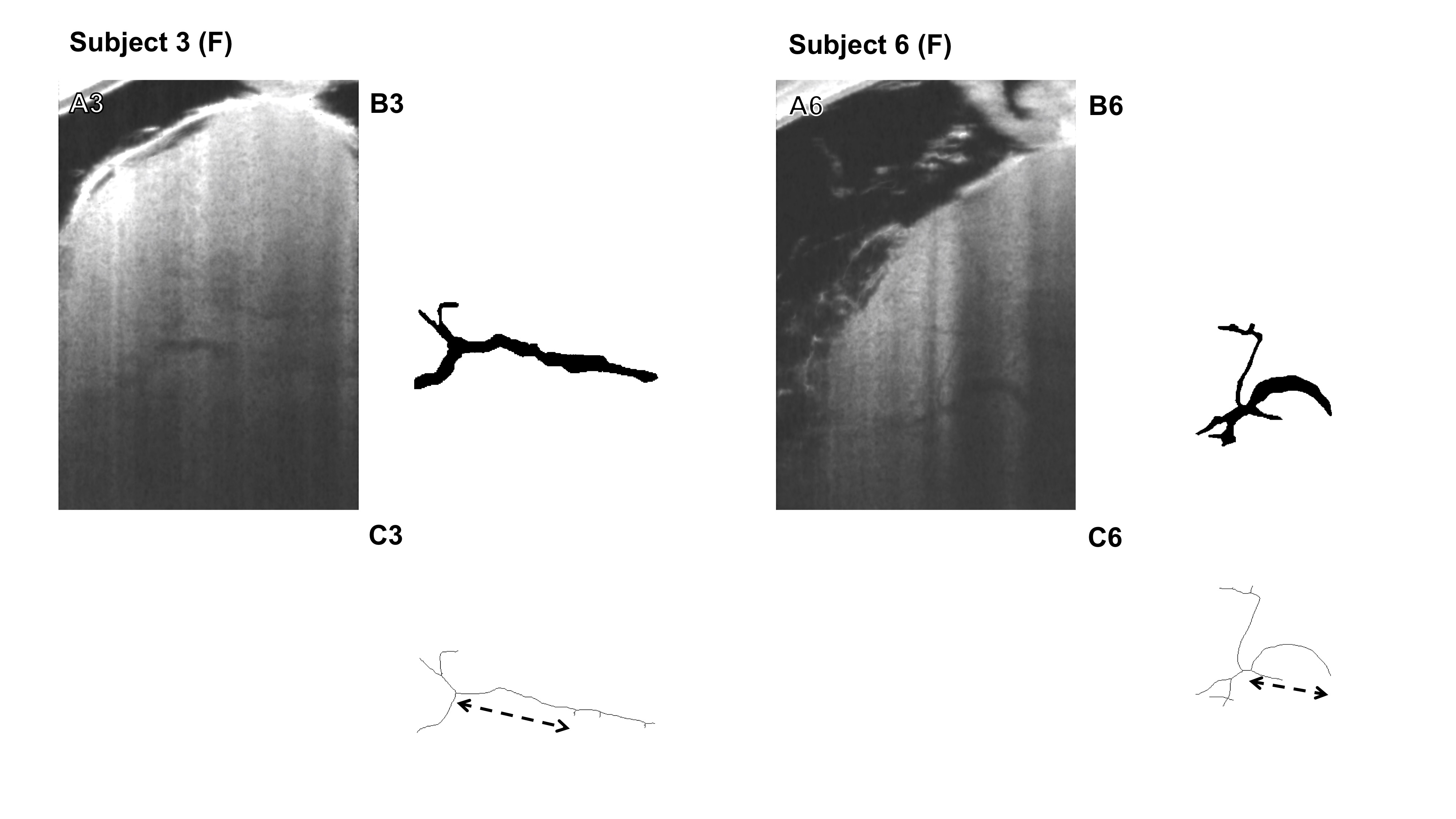

Supplement: Supplementary file 14 — Supplementary Information 9. [file 41598_2020_76468_MOESM14_ESM.jpg]

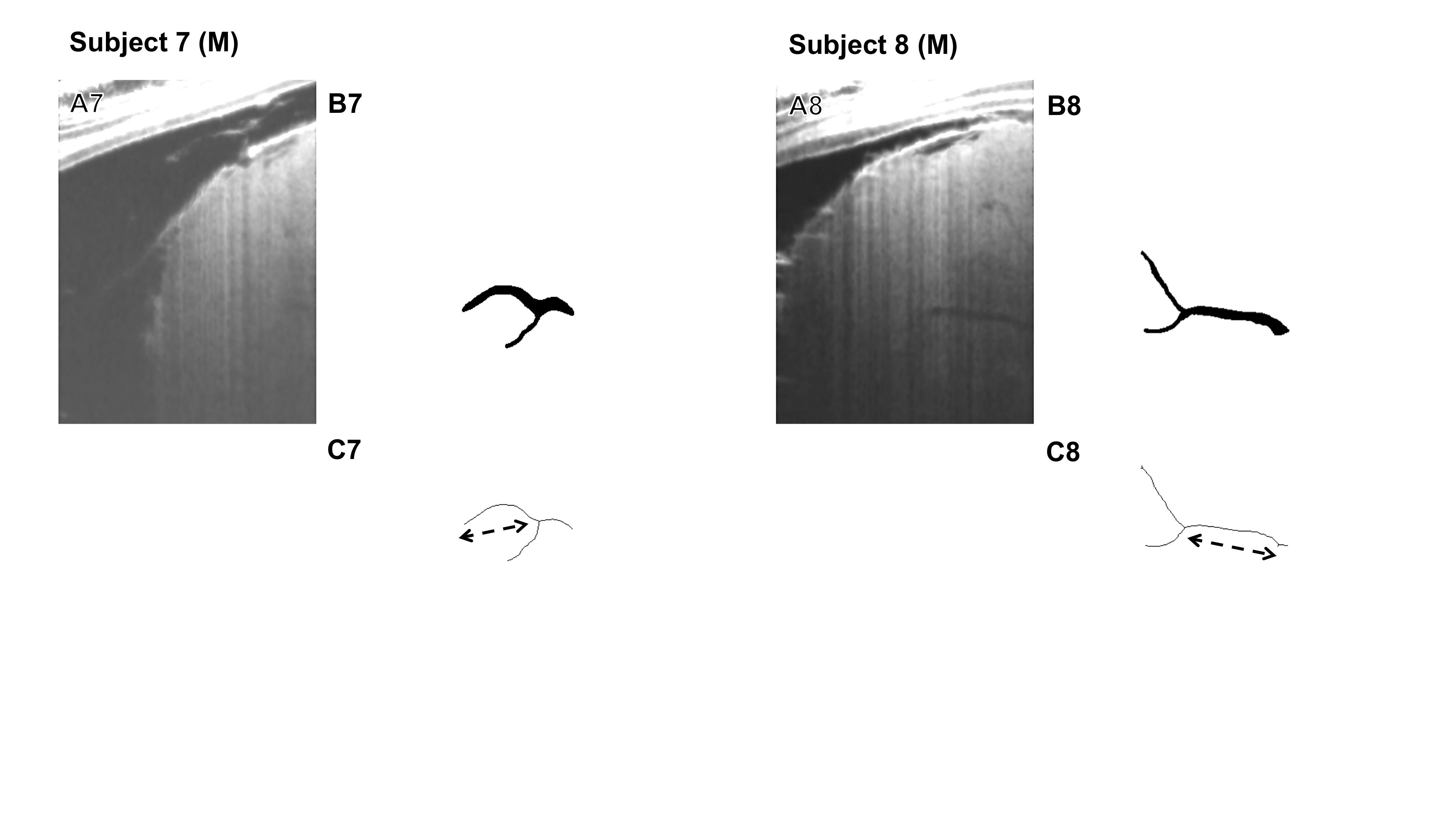

Supplement: Supplementary file 15 — Supplementary Information 10. [file 41598_2020_76468_MOESM15_ESM.jpg]

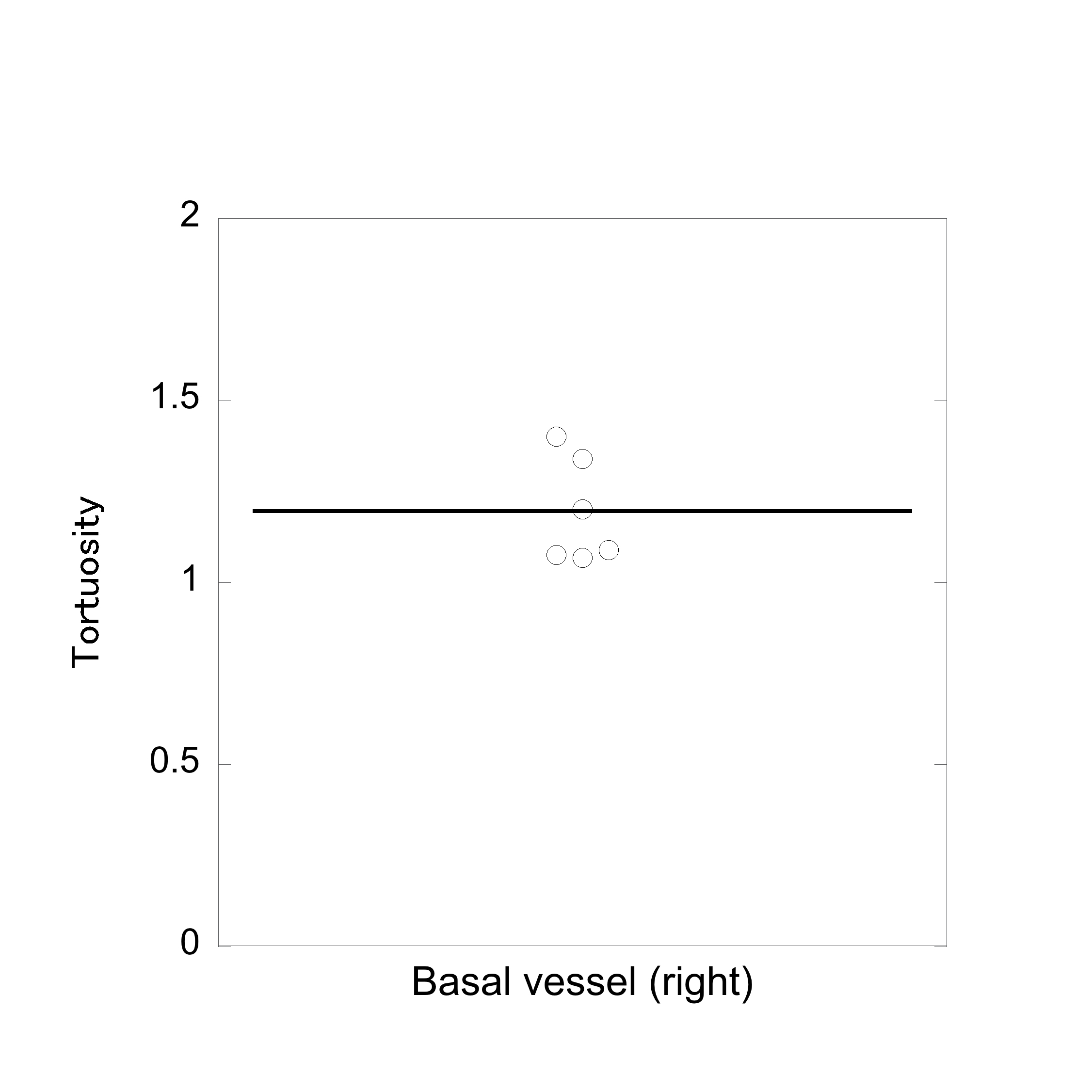

Supplement: Supplementary file 16 — Supplementary Information 11. [file 41598_2020_76468_MOESM16_ESM.jpg]
